# Impaired Cytoskeletal and Membrane Biophysical Properties of Acanthocytes in Hypobetalipoproteinemia – A Case Study

**DOI:** 10.3389/fphys.2021.638027

**Published:** 2021-02-23

**Authors:** Anne-Sophie Cloos, Laura G. M. Daenen, Mauriane Maja, Amaury Stommen, Juliette Vanderroost, Patrick Van Der Smissen, Minke Rab, Jan Westerink, Eric Mignolet, Yvan Larondelle, Romano Terrasi, Giulio G. Muccioli, Andra C. Dumitru, David Alsteens, Richard van Wijk, Donatienne Tyteca

**Affiliations:** ^1^CELL Unit & PICT Imaging Platform, de Duve Institute, UCLouvain, Brussels, Belgium; ^2^Department of Hematology, University Medical Center Utrecht, Utrecht University, Utrecht, Netherlands; ^3^Central Diagnostic Laboratory – Research, University Medical Center Utrecht, Utrecht University, Utrecht, Netherlands; ^4^Department of Vascular Medicine, University Medical Center Utrecht, Utrecht University, Utrecht, Netherlands; ^5^Louvain Institute of Biomolecular Science and Technology, UCLouvain, Ottignies-Louvain-la-Neuve, Belgium; ^6^Bioanalysis and Pharmacology of Bioactive Lipids Research Group, Louvain Drug Research Institute, UCLouvain, Brussels, Belgium

**Keywords:** acanthocytosis, lipidomics, lipid domains, membrane biophysical properties, reactive oxygen species, ceramide, mitochondria, erythropoiesis

## Abstract

Familial hypobetalipoproteinemia is a metabolic disorder mainly caused by mutations in the *apolipoprotein B* gene. In its homozygous form it can lead without treatment to severe ophthalmological and neurological manifestations. In contrast, the heterozygous form is generally asymptomatic but associated with a low risk of cardiovascular disease. Acanthocytes or thorny red blood cells (RBCs) are described for both forms of the disease. However, those morphological changes are poorly characterized and their potential consequences for RBC functionality are not understood. Thus, in the present study, we asked whether, to what extent and how acanthocytes from a patient with heterozygous familial hypobetalipoproteinemia could exhibit altered RBC functionality. Acanthocytes represented 50% of the total RBC population and contained mitoTracker-positive surface patches, indicating the presence of mitochondrial fragments. While RBC osmotic fragility, calcium content and ATP homeostasis were preserved, a slight decrease of RBC deformability combined with an increase of intracellular free reactive oxygen species were observed. The spectrin cytoskeleton was altered, showing a lower density and an enrichment in patches. At the membrane level, no obvious modification of the RBC membrane fatty acids nor of the cholesterol content were detected but the ceramide species were all increased. Membrane stiffness and curvature were also increased whereas transversal asymmetry was preserved. In contrast, lateral asymmetry was highly impaired showing: (i) increased abundance and decreased functionality of sphingomyelin-enriched domains; (ii) cholesterol enrichment in spicules; and (iii) ceramide enrichment in patches. We propose that oxidative stress induces cytoskeletal alterations, leading to increased membrane stiffness and curvature and impaired lipid lateral distribution in domains and spicules. In addition, ceramide- and spectrin-enriched patches could result from a RBC maturation defect. Altogether, the data indicate that acanthocytes are associated with cytoskeletal and membrane lipid lateral asymmetry alterations, while deformability is only mildly impaired. In addition, familial hypobetalipoproteinemia might also affect RBC precursors leading to disturbed RBC maturation. This study paves the way for the potential use of membrane biophysics and lipid vital imaging as new methods for diagnosis of RBC disorders.

## Introduction

Abetalipoproteinemia and familial homozygous hypobetalipo- proteinemia are two very rare metabolic disorders which are clinically indistinguishable (Biemer, 1980). Abetalipoproteinemia is provoked by mutations of both alleles of the *microsomal triglyceride transfer protein* (*MTP*) encoding gene (Calzada et al., 2013). MTP allows the transfer of triglycerides, cholesteryl ester and phospholipids onto the apolipoprotein B (ApoB). Familial hypobetalipoproteinemia is considered as autosomal codominant and most frequently caused by mutations affecting directly the *ApoB* gene leading to truncated forms of the protein (Clarke et al., 2006). In both abetalipoproteinemia and familial homozygous hypobetalipoproteinemia the consequence of gene mutations is failure of release of ApoB-containing lipoproteins, especially chylomicrons and very low density lipoproteins (VLDL) (Burnett and Hooper, 2015). As a result, lipid and lipid soluble vitamin absorption is disturbed, clinically manifesting by extremely low plasma cholesterol and triacylglycerol levels, vitamin A, E, and K levels as well as an almost complete lack of circulating chylomicrons, VLDL and low density lipoproteins (LDL) (Granot and Kohen, 2004). Patients suffering from these diseases present early in life a series of gastroenterological complications including fat malabsorption, steatorrhea, vomiting, abdominal distension and failure to thrive. Later in life, neurological and ophthalmological manifestations, due to lipid soluble vitamin deficiency, as well as non-alcoholic fatty liver disease (NAFLD) become part of the clinical picture (Burnett and Hooper, 2015). For this reason, therapy implies, besides of a strict low fat diet, a life-long supplementation with vitamins A and E (Granot and Kohen, 2004).

Besides abetalipoproteinemia and familial homozygous hypobetalipoproteinemia, a heterozygous form of familial hypobetalipoproteinemia has also been described. Patients are often asymptomatic but frequently associated with NAFLD and in rare cases with neurological disorders (Musialik et al., 2020). Despite the frequently asymptomatic appearance of the heterozygous form of familial hypobetalipoproteinemia, strongly reduced plasma ApoB and LDL-cholesterol levels combined eventually with vitamin E levels below or at the limit of reference values are typical clinical features (Clarke et al., 2006). This heterozygous form is also associated with a low risk of cardiovascular disease (Whitfield et al., 2004).

More surprisingly, acanthocytes (or thorny red blood cells, RBCs) are usually observed on blood smears from patients with abetalipoproteinemia and familial homozygous hypobetalipoproteinemia and to a lesser extent in the heterozygous form (Whitfield et al., 2004; Cuerq et al., 2018). Acanthocytes are RBCs with few membrane projections varying in length and presenting a non-uniform distribution along the RBC surface, in contrast to echinocytes which exhibit more regular membrane projections that are evenly distributed on the RBC membrane. Acanthocytes are, however, not specific to the above mentioned metabolic disorders but are also associated with a series of other pathologies and disorders including chorea-acanthocytosis, McLeod phenotype, In(Lu) phenotype, hereditary spherocytosis with a β-spectrin deficiency, alcoholic cirrhosis, uremia, vitamin E deficiency, anorexia nervosa and hypothyroidism (Wong, 2004).

Such acanthocyte shape could lead to alteration of RBC deformability, which mainly results from the RBC typical biconcave shape as determined by a membrane surface area to cytoplasmatic volume excess. Besides, RBC deformability is also determined by a finely regulated cytoplasmic viscosity controlled by hemoglobin (Hb) concentration and a cytoskeleton composed of a meshwork of spectrin tetramers linked to the membrane by the 4.1R- and ankyrin-based anchorage complexes (Salomao et al., 2008; Baines, 2010). RBC deformation also depends on the intracellular ATP content, the antioxidant defense, the ion balance and subsequent volume control regulated by ion channels, symporters, antiporters and pumps. Among ion channels, the mechanosensitive non-selective cation channel PIEZO1 has been recently identified as the link between mechanical forces, calcium influx and RBC volume homeostasis. The calcium-activated K^+^ channel (named Gardos), the Cl^–^/HCO_3_^–^ antiporter Band3 and the plasma membrane calcium ATPase pump (PMCA) are also essential for the RBC homeostasis. For instance, the transient increase in intracellular calcium upon deformation activates Gardos channels, leading to cell dehydration and favoring local membrane:cytoskeleton uncoupling (Bogdanova et al., 2013; Cahalan et al., 2015; Lew and Tiffert, 2017; Kaestner et al., 2020). In addition, RBC membrane lipid composition and distribution are also important for RBC deformation. Actually, the RBC plasma membrane exhibits a high cholesterol content as compared to other cells and shows lipid clustering in domains (Carquin et al., 2016). Three types of lipid domains coexist at the RBC outer membrane leaflet (D’Auria et al., 2013; Carquin et al., 2014, 2015; Leonard et al., 2017b; Conrard et al., 2018). The first, associated with high-curvature membrane areas, is mainly enriched in cholesterol and gathers and/or stabilizes high curvature membrane areas upon RBC deformation. The second and third domains are associated with low-curvature membrane areas. They are, respectively, enriched in ganglioside GM1, phosphatidylcholine and cholesterol (hereafter named GM1-enriched domains) and in sphingomyelin, phosphatidylcholine and cholesterol (hereafter named sphingomyelin-enriched domains). Both GM1- and sphingomyelin-enriched domains seem to participate in calcium exchanges during RBC deformation as GM1-enriched domain abundance increases upon calcium influx after PIEZO1 activation while sphingomyelin-enriched domain abundance increases during calcium efflux (Leonard et al., 2017b; Conrard et al., 2018). A fourth type of lipid domain, mainly enriched in ceramide and associated to the membrane inner leaflet, has been recently identified (Cloos et al., 2020).

In the present study, we asked whether, to what extent and how acanthocytes from a patient with heterozygous familial hypobetalipoproteinemia (pHypoβ) could exhibit alteration of RBC deformability. For this purpose, the RBCs of the patient, who presented a high number of acanthocytes on blood smears, were analyzed for the main features associated with RBC deformability, i.e., RBC morphology, the spectrin cytoskeleton integrity, the intracellular ATP content, the antioxidant defense, the ion balance and the RBC membrane lipid composition and biophysical properties.

The experimental approaches were chosen based on our previous work (Carquin et al., 2014, 2015; Leonard et al., 2017a; Pollet et al., 2018, 2020; Cloos et al., 2020): (i) for RBC morphology: blood smears, optical microscopy of living RBCs and scanning electron microscopy (SEM) of fixed RBCs; (ii) for RBC deformability: osmotic fragility through Hb release and deformability upon shear stress by ektacytometry; (iii) for cytoskeleton integrity: spectrin confocal imaging; (iv) for intracellular ATP content: a biochemical assay; (v) for the antioxidant defense: extent of oxidative stress through measurement of free intracellular reactive oxygen species (ROS) content, lipid peroxidation and Hb oxidation; (vi) for the ion balance: intracellular calcium content evaluated by flow cytometry; (vii) for membrane lipid composition: determination of fatty acids (FA), cholesterol, sphingolipids and phospholipids content; and (viii) for membrane biophysical properties: evaluation of elastic modulus by atomic force microscopy (AFM), curvature by optical microscopy of living RBCs in suspension, transversal asymmetry by phosphatidylserine (PS) surface exposure and lateral asymmetry by vital confocal imaging using validated fluorescent lipid analogs or toxin fragments specific to endogenous lipids. We finally explored whether impairments resulted from defects during RBC maturation or from acceleration of aging. Our findings improve our understanding of hypobetalipoproteinemia and might be extended to other RBC diseases.

## Materials and Methods

### Blood Collection and Preparation

The study was approved by the Medical Ethics Committee of UCLouvain and University Medical Center Utrecht (Study 17–450). After informed consent, blood from the patient and 4 healthy volunteers was collected by venipuncture into K^+^/EDTA-coated tubes at the University Medical Center Utrecht. Whenever possible, healthy donors were selected to be age- and gender-matched. After collection, the tubes were transferred to the research laboratory at UCLouvain. Two splenectomised healthy donors were included in the study. Before experiments, RBCs were collected through 10-fold blood dilution in a glucose- and HEPES-containing medium [Dulbecco’s modified eagle medium (DMEM), Invitrogen]. Diluted blood was centrifugated at 200 *g* for 2 min, the supernatant removed and RBCs suspended in medium. RBCs were washed a second time by centrifugation at 200 *g* for 2 min and resuspended, as in Cloos et al. (2020). The number of RBCs used for experiments were as follows: (i) Hb release, 12.5 × 10^6^; (ii) intracellular ATP content, 5 × 10^6^; (iii) calcium and ROS contents, 15 × 10^6^; (iv) lipidomic and FA analyses, 2.5–6 × 10^8^; (v) PS surface exposure, 0.5 × 10^6^; (vi) cholesterol assay, 37.5 × 10^6^; and (vii) fluorescence imaging, 12.5 × 10^6^.

### RBC Chemical Treatments

Chemical treatments were performed on washed RBCs. To activate calcium entry through PIEZO1, RBCs were incubated with 0.1 μM Yoda1 (Bio-Techne) for 30 s at RT. To deplete the intracellular calcium content, RBCs were preincubated in a calcium-free medium containing 1mM EGTA (Sigma-Aldrich) for 10 min at RT and maintained during the experiment. To induce oxidative stress, RBCs were submitted to 100 mM H_2_O_2_ for 60 min at 37°C.

### RBC Morphology Determination

RBC morphology was determined (i) on blood smears, (ii) upon suspension of living RBCs in μ-dish IBIDI chambers, (iii) by SEM on fixed RBCs, and (iv) after immobilization of living RBCs on poly-L-lysine (PLL)-coated coverslips. For the RBCs in suspension, washed RBCs were diluted 24-fold in DMEM, deposed in μ-dish IBIDI chambers and observed with a wide-field fluorescence microscope (Observer.Z1; plan-Apochromat 100× 1.4 oil Ph3 objective). For SEM microscopy experiments, RBCs were prepared and analyzed exactly as in Cloos et al. (2020). For RBC immobilization, PLL was deposed on coverslips for 30 min at 37°C and then washed. RBCs were then spread on the PLL-coated coverslip during 4 min and the coverslip was then placed upside down in a medium-filled LabTek chamber (Fisher Scientific). RBCs were observed with the fluorescence microscope Observer.Z1 as above.

### RBC Hemoglobin Release Measurement

Washed RBCs were incubated for 10 min at RT in isotonic (320 mOsm, DMEM) or increasingly hypotonic media (264–0 mOsm, DMEM mixed with water to obtain the desired osmolarity) and then pelleted by centrifugation at 200 *g* for 2 min. Supernatants and pellets broken with 0.2% (*w*/*v*) Triton X-100 were both assessed for Hb at 450 nm in 96-well plates (SpectraCount^TM^, Packard BioScience Co.). Hb release in the supernatant was expressed as percentage of the total Hb present in the sample.

### Ektacytometry

Deformability of RBCs was measured with the Laser Optical Rotational Red Cell Analyzer (Lorrca, RR Mechatronics, Zwaag, Netherlands). In this ektacytometer, RBCs are exposed to shear stress in a viscous solution (Elon-Iso, RR Mechatronics), forcing the cells to elongate in an elliptical shape. The diffraction pattern, that is generated by a laser beam, is measured by a camera. The vertical axis (A) and the horizontal axis (B) of the ellipse are used to calculate the elongation index (EI) by the formula (*A* − *B*)/(*A* + *B*). The EI reflects the deformability of the total population of RBCs. Two different forms of ektacytometry were used: osmotic gradient ektacytometry and cell membrane stability test (CMST). Osmotic gradient ektacytometry measurements of RBCs were obtained using the osmoscan module, according to the manufacturer’s instructions and as described elsewhere (Da Costa et al., 2016; Lazarova et al., 2017). Briefly, 250 μl whole blood was standardized to a fixed RBC count of 1000^∗^10^6^ and mixed with 5 ml of Elon-Iso. RBCs in polyvinylpyrrolidone (PVP) were exposed to an osmolarity gradient from approximately 60–600 mOsmol/L, whereas shear stress was kept constant (30 Pa). The CMST was performed using the CMST module on the ektacytometer. To perform a CMST, 50 μl of whole blood was standardized to a fixed RBC count of 200^∗^10^6^ and mixed with 5 ml of Elon-Iso. In the CMST, RBCs are exposed to a shear stress of 100 Pa for 3,600 s (1 h) while the EI is continuously measured. The change in the elongation index (ΔEI) was calculated by determining the median of the first and the last 100 s of the CMST and subsequently calculating the difference between the medians. The ΔEI depicts the capacity of the RBCs to shed membrane and resist shear stress.

### Intracellular ATP Measurement

The intracellular ATP level was determined by a chemiluminescence assay kit (Abcam) as described in Conrard et al. (2018) and Cloos et al. (2020). ATP levels were then reported to the corresponding Hb content measured by spectrophotometry.

### Intracellular Calcium Determination

Intracellular calcium content was determined by RBC labeling with Fluo4-AM (Invitrogen) as in Cloos et al. (2020). Labeled RBCs were then either analyzed by flow cytometry (FACSVerse, BD Biosciences) or fluorimetry (GloMax; Promega; λ_*exc*_ of 490 nm and λ_*em*_ of 520 nm). Flow cytometry data, obtained by medium flow rate on 10.000 events, was analyzed with the software FlowJo to determine the median fluorescence intensity (MFI) of analyzed RBCs. Fluorimetry data were normalized to the global Hb content determined spectrophotometrically as above.

### RBC Fatty Acid Content

RBC FA composition was assessed exactly as described in Cloos et al. (2020).

### RBC Lipid Composition

The cholesterol content of washed RBCs was determined with a fluorescent assay kit (Invitrogen) as in Cloos et al. (2020) and reported to the global Hb content as described above. The membrane composition in phospholipids, lysophospholipids, sphingolipids and oxysterols was analyzed by lipidomics according to Guillemot-Legris et al. (2016) and Mutemberezi et al. (2016), as described in Cloos et al. (2020).

### Oxidative Stress Measurements

Intracellular free ROS were assessed by RBC labeling with 2,7-dichlorodihydrofluoresceindiacetate (H_2_DCFDA) in Krebs–Ringer–Hepes (KRH) solution for 30 min at 37°C. MFI of the whole RBC population was acquired as explained above for Fluo4-AM. Lipid peroxidation was evaluated by measurement of malondialdehyde (MDA) with a lipid peroxidation assay kit (Abcam) according to the high sensitivity protocol as in Cloos et al. (2020). Methemoglobin (metHb) was analyzed in RBC lysates, produced through repeated freeze–thaw cycles, with a sandwich Elisa kit (Lifespan Biosciences) as described in Cloos et al. (2020). Both MDA levels and metHb contents were reported to the global Hb content.

### Membrane Lipid Imaging on Living RBCs

Endogenous cholesterol was evidenced through living RBC labeling with mCherry-Theta toxin fragment followed by immobilization on PLL-coated coverslips (Cloos et al., 2020). To visualize GM1 ganglioside, sphingomyelin and ceramide, living RBCs were spread onto PLL-coated coverslips and then labeled by trace insertion in the plasma membrane of BODIPY fluorescent analogs of those lipids (Invitrogen) as described in Conrard et al. (2018). Coverslips with living immobilized RBCs were placed in medium-filled LabTek chambers and observed with the wide-field fluorescence microscope (Observer.Z1; plan-Apochromat 100× 1.4 oil Ph3 objective).

### Mitochondria Fragment Imaging on Living RBCs

To determine the presence of mitochondria fragments in RBCs, washed RBCs were labeled with 100 nM mitoTracker (Invitrogen) at RT for 30 min, washed, placed on PLL-coated coverslips and observed with the wide-field fluorescence microscope Observer.Z1.

### Membrane Curvature on Living RBCs

RBCs were placed into IBIDI chambers as described above for RBC morphology in suspension. Microscopy images were then analyzed with the last version of Shape Analysis by Fourier Descriptors computation plugging for ImageJ as in Leonard et al. (2017b) and Pollet et al. (2020).

### Membrane Transversal Asymmetry on Living RBCs

Exposure of PS was assessed by RBC labeling with Annexin-V coupled to FITC (Invitrogen) and analyzed by flow cytometry as in Cloos et al. (2020). The proportion of PS-exposing RBCs was then determined with the FlowJo software by positioning the cursor at the edge of the healthy RBC population.

### Force Distance-Based Atomic Force Microscopy on Living RBCs

AFM experiments were performed with a Bioscope Resolve AFM (Bruker) at ∼25–30°C in DMEM. PeakForce QNM Live Cell probes (Bruker) with spring constants of 0.10 ± 0.02 N m^–1^ and tip radius of curvature of 65 nm were used. The spring constant of the cantilevers was calibrated with a vibrometer (OFV-551, Polytec, Waldbronn) by the manufacturer. The pre-calibrated spring constant was used to determine the deflection sensitivity (Schillers et al., 2017) using the thermal noise method (Hutter and Bechhoefer, 1993) before each experiment. In fast indentation experiments (Dumitru et al., 2018), the AFM was operated in PeakForce QNM mode and Force-distance (FD)-based multiparametric maps were acquired using a force setpoint of 300 pN. The AFM cantilever was oscillated vertically at 0.25 kHz with a peak-to-peak oscillation amplitude of 500 nm. Height and Young’s modulus maps were recorded using a scan rate of 0.2 Hz and 256 pixels per line. Slow indentation experiments were performed in Force-Volume (FV) mode. Individual FD curves were recorded in contact mode on the RBC surface with a force setpoint of 300 pN, using ramp speeds of 2 μm s^–1^ for a 2 μm ramp size. Measurements were performed in the central region of RBCs to avoid substrate effects. Indentations ranged between 300 nm and 1 μm depending on cell type.

### RBC Spectrin Immunofluorescence

Immunolabeling of spectrin was performed as in Cloos et al. (2020) and Pollet et al. (2020). All coverslips were mounted with Dako and examined with a Zeiss Cell Observer Spinning Disk (COSD) confocal microscope using a plan-Apochromat 100× NA 1.4 oil immersion objective and the same settings for illumination.

### Image Analysis, Data Quantification and Statistical Analyses

RBC membrane area, RBC curvature and spectrin intensity/occupation were determined using the Fiji software. Lipid domain abundance was determined by manual counting and expressed by reference to the hemi-RBC projected area. The proportion of spiculated RBCs and of RBCs presenting lipid- or mitoTracker-enriched vesicles, patches or spicules was also assessed by manual counting on fluorescence images. For AFM, at least 64 FD curves were recorded per cell and analyzed using the Nanoscope Analysis v9.1 software (Bruker). Hertz model was used to extract Young’s modulus values from individual FD curves. Whole RBC elasticity was analyzed from Slow indentation experiments in FD-AFM mode, by fitting the repulsive part of FD curves with the Hertz model. To analyze the contribution of the plasma membrane and cytoskeleton to the cellular mechanical behavior, two different fit ranges were defined in the repulsive part of FD curves obtained in Fast indentation experiments (PeakForce QNM mode). Plasma membrane elastic contribution was estimated for indentations δ < 50 nm and cytoskeleton elasticity was extracted from δ between 50 and 100 nm. Data are expressed as means ± SEM when the number of independent experiments was *n* ≥ 3 or means ± SD if *n* ≤ 2. For all experiments, statistical tests were performed only when *n* ≥ 3. Tests were non-parametrical Mann–Whitney test or Kruskal–Wallis followed by Dunn’s comparison test, except for AFM and membrane curvature data for which two sample *t*-tests were applied as RBCs were analyzed individually and data obtained for all RBCs are presented. To evaluate the effect of chemical agents, the paired data were analyzed by paired non-parametrical tests (Wilcoxon matched-pairs signed rank tests). ns, not significant; ^∗^*p* < 0.05, ^∗∗^*p* < 0.01, ^****^p < 0.0001.

## Results

### Case Presentation

**FIGURE 1:**
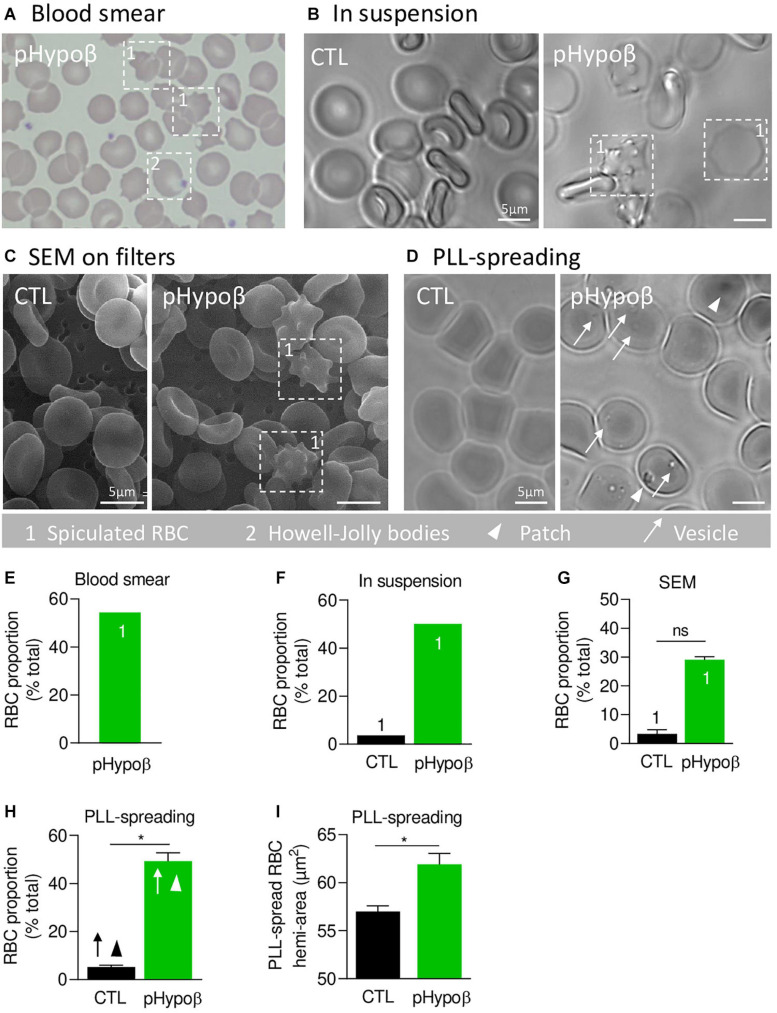
A high proportion of acanthocytes is visible on pHypoβ blood smears and RBCs in suspension while patches and small vesicles are evidenced upon RBC spreading. The morphology of RBCs of the patient with hypobetalipoproteinemia (pHypoβ; green columns) was compared to those of healthy donors (CTL; black columns), either on blood smear **(A)**, on RBCs in suspension **(B,C)**, or upon spreading on poly-L-lysine (PLL)-coated coverslips **(D)**. Then, the proportion of RBCs with spicules (indicated with 1; **E–G**), patches (arrowheads, **H**) and/or vesicles (arrows, **H**) and the RBC membrane area were determined **(I)**. **(A)** May–Grünwald Giemsa-stained blood smear. 1, spiculated RBCs; 2, Howell-Jolly bodies. **(B)** Light microscopy of living RBCs in suspension in IBIDI chambers. Images are representative of 2 independent experiments. **(C)** Scanning electron microscopy of glutaraldehyde-fixed RBCs on filters. Images are representative of 2 experiments with 3 filters each. **(D)** Light microscopy of living RBCs spread on PLL-coated coverslips. Images are representative of at least 10 experiments. **(E–G)** Quantification of the abundance of spiculated RBCs, expressed as % of total RBCs. Data are means from 1 experiment for **(E,F)** and means ± SEM of 3–4 filters in **(G)**. Mann–Whitney test; ns, not significant. **(H)** Quantification of the proportion of PLL-spread RBCs presenting patches and/or vesicles. Data are means ± SEM of 3–6 independent experiments. Mann–Whitney test; *p < 0.05. **(I)** Quantification of the membrane surface area of PLL-spread RBCs. Data are means ± SEM of 6–7 independent experiments. Mann–Whitney test; *p < 0.05.

The patient pHypoβ is a 49 year-old adult. Due to idiopathic thrombocytopenic purpura, his spleen has been removed at the age of 17 as a curative treatment. He has no additional treatment. The transaminases are increased (Supplementary Table 1) but he has no anemia and is otherwise healthy. He exhibits since more than 30 years a high number of acanthocytes on blood smears (Figure 1A), which was suspected to result from hypobetalipoproteinemia. The patient also presented a vitamin E level close to the lower reference limit (Supplementary Table 1). Despite of acanthocytosis, reticulocyte count, RBC mean corpuscular volume and Hb levels are within the normal range (Supplementary Table 1).

### pHypoβ Presents a Nucleotide Deletion in *APOB* Resulting in a Truncated ApoB

Next-Generation Sequencing of pHypoβ revealed a heterozygous single nucleotide deletion in *APOB*, the gene encoding ApoB. It concerns the deletion of adenosine at nucleotide 2,534 in exon 17, which leads to a shift in the reading frame and consequent premature end of translation at residue 862 (Human Gene Mutation Database, CD051293). Nonsense-mediated mRNA decay likely will prevent translation of such mutant *APOB* mRNA into a severely truncated protein. This mutation has already been identified (Fouchier et al., 2005), but consequences for RBC morphology, cytoskeletal and biophysical properties as well as functionality were not evaluated.

### pHypoβ Exhibits a High Proportion of Acanthocytes

We started by confirming the presence of spiculated RBCs detected on a blood smear (Figure 1A) by optical microscopy of living RBCs in suspension and by SEM of fixed RBCs on filters (Figures 1B,C). Quantification indicated that spiculated RBCs represented ∼50% of all pHypoβ RBCs in blood smear and optical microscopy images and ∼30% in electron microscopy images while they accounted only for a minority of RBCs from healthy donors (Figures 1E–G). Discrepancies regarding the proportion of spiculated RBCs detected by electron and optical microscopy could result from the shear stress induced by filtration during RBC preparation for SEM, eventually leading to hemolysis of spiculated RBCs.

When RBCs were spread on PLL-coated coverslips, spiculated RBCs could still be distinguished from other RBCs as they presented big dark patches and smaller clearer vesicles at their surface (arrowheads and arrows at Figure 1D). The proportion of such RBCs was significantly increased in pHypoβ (Figure 1H) and corresponded exactly to the proportion determined above for non-spread RBCs. RBC morphological alterations were accompanied by an increased membrane area of spread RBCs (Figure 1I) and by the presence of Howell-Jolly bodies on blood smears (Figure 1A), compatible with splenectomy. In contrast, the increase of membrane area did not result from splenic absence since a healthy splenectomised donor did not show such a membrane surface area increase (Supplementary Figure 1A).

### pHypoβ RBCs Show no Evidence for Increased Osmotic Fragility nor Modifications of ATP and Calcium Homeostasis but a Reduced Ability to Shed Membrane Upon Shear Stress

**FIGURE 2:**
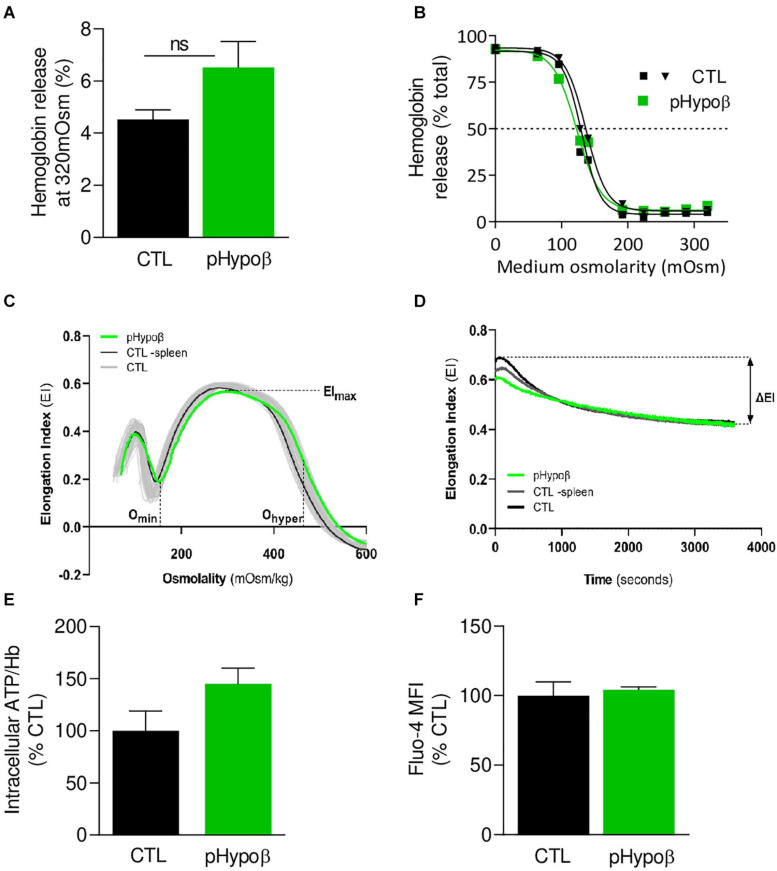
The extent of hemoglobin release and the intracellular calcium and ATP levels are preserved in pHypoβ RBCs while deformability upon shear stress is slightly decreased. RBCs from healthy donors (black and gray in panel **C**) or pHypoβ (green) were evaluated for osmotic fragility **(A,B)**, deformability **(C,D)**, intracellular ATP **(E)** and calcium content **(F)**. **(A,B)** RBC osmotic fragility. RBCs were incubated in isotonic **(A)** or increasingly hypotonic **(B)** media and then centrifugated. Hemoglobin (Hb) in supernatants and in RBC pellets was assessed spectrophotometrically to determine hemolysis. The horizontal dotted line in **(B)** indicates the medium osmolarity at which 50% of the RBCs were lysed. Data are means ± SEM of 4 independent experiments for **(A)** and are representative for 2 independent experiments in **(B)**. Mann–Whitney test; ns, not significant. **(C,D)** RBC deformability. **(C)** Osmotic gradient ektacytometry curve and derived EI_*max*_, *O*_*min*_, and *O*_*hyper*_ parameters, which, respectively, reflect membrane surface area, surface area-to-volume ratio and cellular hydration. pHypoβ RBCs (green curve) were compared to healthy controls (gray curves obtained from 25 healthy subjects) and a healthy splenectomized control (black curve). **(D)** Cell membrane stability test (CMST) curve and the derived ΔEI parameter which depicts the capacity of the RBCs to shed membrane and resist shear stress. Data are representative of 2 experiments in **(C)** and 1 experiment in **(D)**. **(E)** Intracellular ATP. ATP levels were determined with a kit based on the activity of the firefly luciferase in presence of ATP and the consequent light emission in presence of luciferin. Intracellular ATP levels were normalized to Hb and expressed as percentage of the CTL RBCs. Data are means ± SD of triplicates from 1 experiment. **(F)** RBC calcium content. RBCs were labeled with the non-fluorescent Fluo4-AM which is transformed in RBCs into the fluorescent Fluo4 after de-esterification and interaction with calcium ions. Labeled RBCs were analyzed by flow cytometry for median fluorescence intensity (MFI) and then expressed as percentage of CTLs. Data are means ± SD of triplicates from 1 experiment.

Despite the high proportion of pHypoβ RBCs with a modified morphology, their resistance to hemolysis was similar to healthy controls (splenectomised or not), as shown by Hb release after RBC incubation in isotonic and increasingly hypotonic media (Figures 2A,B and Supplementary Figure 1B). Nevertheless, the more sensitive osmotic gradient ektacytometry test revealed that the *O*_*min*_ value was slightly increased in pHypoβ (153 vs. 125–148 in healthy donors), indicative of a slight decrease in surface area-to-volume ratio and a slight increase in osmotic fragility (Figure 2C). Moreover, the EI_*max*_, and therefore the surface area, were slightly decreased in pHypoβ RBCs (0.566 vs. 0.585–0.607). This decrease *a priori* contrasted with the increased surface area determined by optical microscopy on spread RBCs which could result from the increase of the outer leaflet membrane area specifically (Figure 1I). Those changes did not result from splenectomy since all osmotic gradient ektacytometry-derived parameters were normal in the healthy splenectomized control (Figure 2C). To further investigate pHypoβ RBC deformability, the CMST was performed to assess the RBC ability to shed membrane when exposed to prolonged supraphysiological shear stress force. This is a physiological property of healthy RBCs and the decrease in EI that occurs under these circumstances is proposed to be a measure of membrane health. pHypoβ RBCs showed a ΔEI of −0.144, which was considerably less than the ΔEI of −0.187 from the healthy splenectomized control, and even more less than the ΔEI of −0.208 seen in healthy donors (Figure 2D). These results indicated that splenectomy on itself was accompanied with loss of the ability to shed membrane upon shear stress, but that this loss was more pronounced in pHypoβ RBCs. We also evaluated the intracellular contents in ATP and calcium, two key parameters for RBC functionality and deformation (Bogdanova et al., 2013; Conrard et al., 2018). The intracellular ATP content was slightly increased but the calcium content remained unchanged (Figures 2E,F).

### pHypoβ RBCs Exhibit Normal Membrane Fatty Acid Profile and Cholesterol Content but Increased Ceramide Species

**FIGURE 3:**
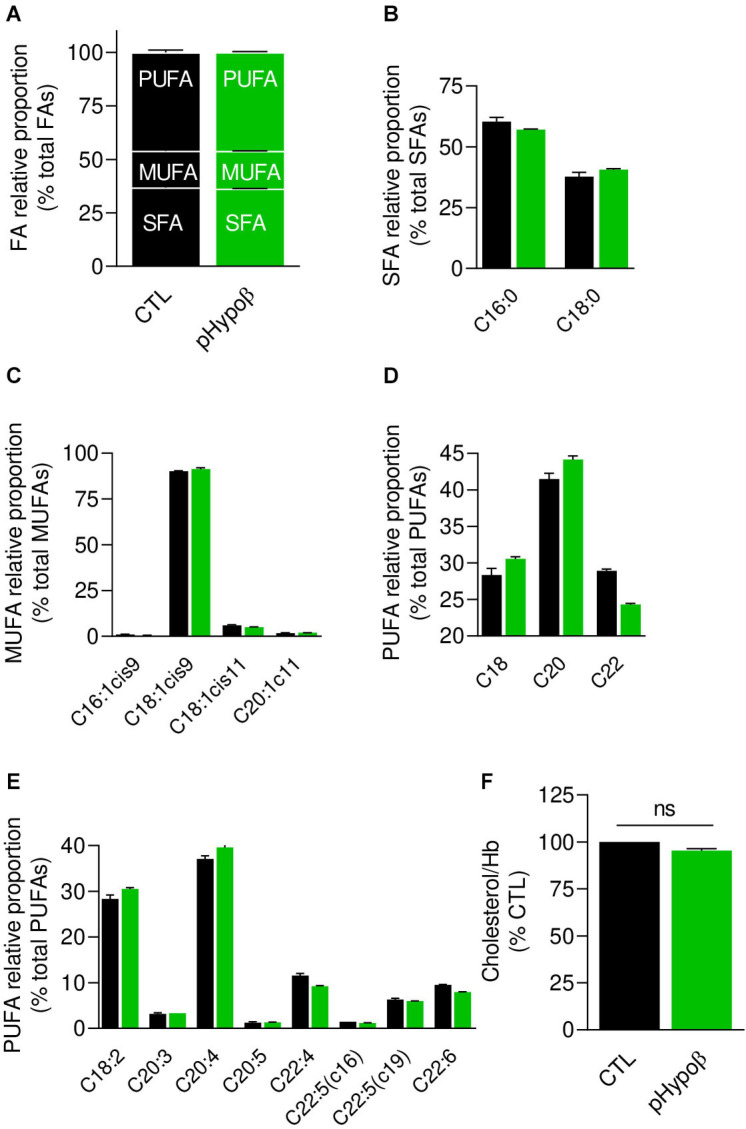
Membrane fatty acid and cholesterol contents are largely preserved in pHypoβ RBCs. RBCs from healthy donors (black columns) or pHypoβ (green columns) were evaluated for membrane fatty acid (FA) content **(A–E)** and cholesterol level **(F)**. **(A–E)** RBC FA composition. Lipids were extracted from isolated RBCs and prepared for gas chromatography to analyze FA content. **(A)** Relative proportion of saturated (SFA), monounsaturated (MUFA) and polyunsaturated (PUFA) FAs expressed as percentage of total FAs. **(B)** Relative proportion of the two major SFAs (C16:0 and C18:0). **(C)** Relative proportion of the four major MUFAs (C16 and C18 with one double bond on position 9 and C18 and C20 with one double bound on position 11). **(D)** Relative proportion of PUFA according to the carbon chain length (chains of 18, 20, or 22C). **(E)** Relative proportion of the major PUFAs (chains of 18–22C and 2–6 double bonds). All data are means ± SD of triplicates from 1 experiment. **(F)** Cholesterol content. Lysed RBCs were evaluated for their cholesterol content through a fluorescent assay kit which uses several enzymatic reactions starting with cholesterol and ending with the transformation of Amplex Red into fluorescent resofurin. Cholesterol content was normalized to Hb content and expressed as percentage of the CTL RBCs. Data are means ± SEM of 3 independent experiments. Mann–Whitney test; ns, not significant.

**FIGURE 4:**
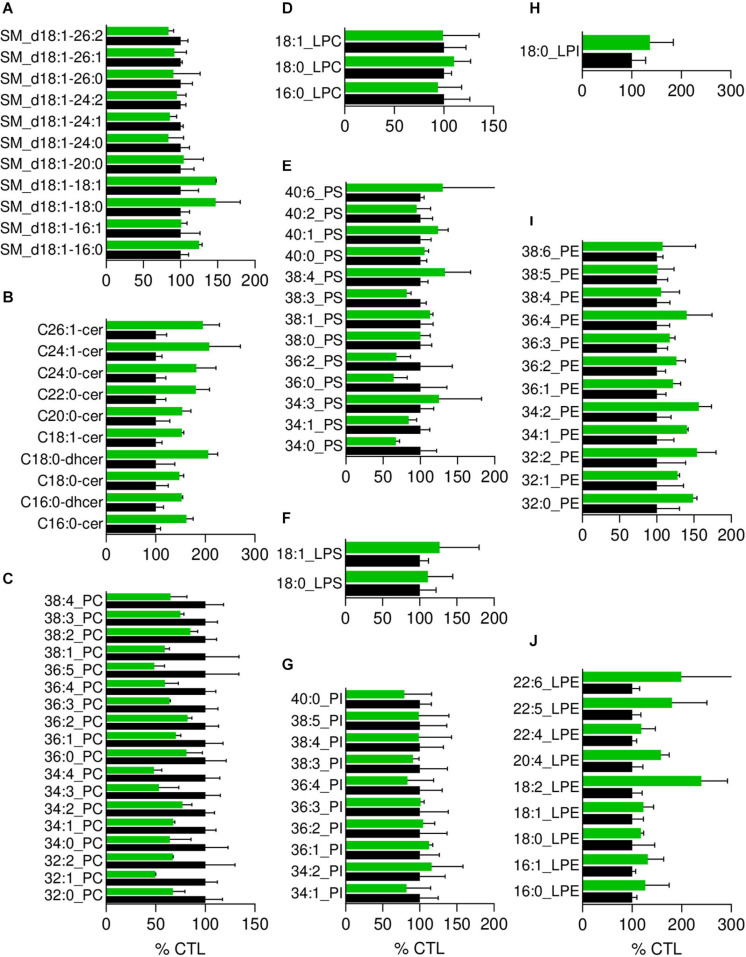
The most obvious membrane lipid changes in pHypoβ RBCs are an increase of ceramide species and a decrease of phosphatidylcholine species. Lipid species were assessed by HPLC-MS on washed, lysed and lipid-extracted RBCs. Content in sphingomyelin (d18:1 D-erythro-sphingosine backbone, SM; **A**), ceramide (Cer) and dihydroceramide (dhcer; **B**), phosphatidylcholine (PC; **C**), lysophosphatidylcholine (LPC; **D**), phosphatidylserine (PS; **E**), lysophosphatidylserine (LPS; **F**), phosphatidylinositol (PI; **G**), lysophosphatidylinositol (LPI; **H**), phosphatidylethanolamnine (PE; **I**) and lysophosphatidylethanolamine (LPE; **J**). Results are expressed as percentage of controls (CTL, mean of 4 donors). Data are means ± SD of 2 independent experiments.

As acanthocytotic RBCs in abetalipoproteinemia and homozygous familial hypobetalipoproteinemia are associated with profound alterations of membrane lipid composition (Biemer, 1980; Barenholz et al., 1981; Gheeraert et al., 1988), we then determined whether RBC morphology alterations in pHypoβ could be associated with modified membrane composition in FAs (Figures 3A–E), cholesterol (Figure 3F), phospholipids and/or sphingolipids (Figure 4). The relative proportion of saturated (SFA), monounsaturated (MUFA) and polyunsaturated (PUFA) FAs was unchanged in pHypoβ RBCs (Figure 3A). Closer examination of major SFAs and MUFAs did not reveal more changes (Figures 3B,C). For PUFAs, however, the proportion of long chain C22 PUFAs appeared to decrease in favor of PUFAs with shorter C18 or C20 chains (Figure 3D). The increase of C18 and C20 PUFAs appeared to result from the higher relative contents in linoleic (C18:2) and arachidonic (C20:4) acids (Figure 3E).

Since the plasmatic cholesterol content was decreased in pHypoβ (Supplementary Table 1), we then evaluated the RBC membrane cholesterol content. Surprisingly, it was not modified in the patient as compared to the healthy donors (Figure 3F). Sphingomyelin species were also largely maintained (Figure 4A) whereas all ceramide and dihydroceramide species, whatever their fatty acid length and unsaturation number, were increased by 1.5- to 2-fold in pHypoβ (Figure 4B). Among phospholipids, no obvious change was detected for PS and phosphatidylinositol (PI) but phosphatidylcholine (PC) species were decreased and phosphatidylethanolamine (PE) species were very slightly increased (Figures 4C,E,G,I). In agreement with the heightened proportion of PE, four lysoPE species were also increased. The other lysophospholipids remained unaffected in pHypoβ (Figures 4D,F,H,J).

### pHypoβ RBCs Have Increased Free Reactive Oxygen Species but No Modification of Phospholipid, Cholesterol and Hemoglobin Oxidation

**FIGURE 5:**
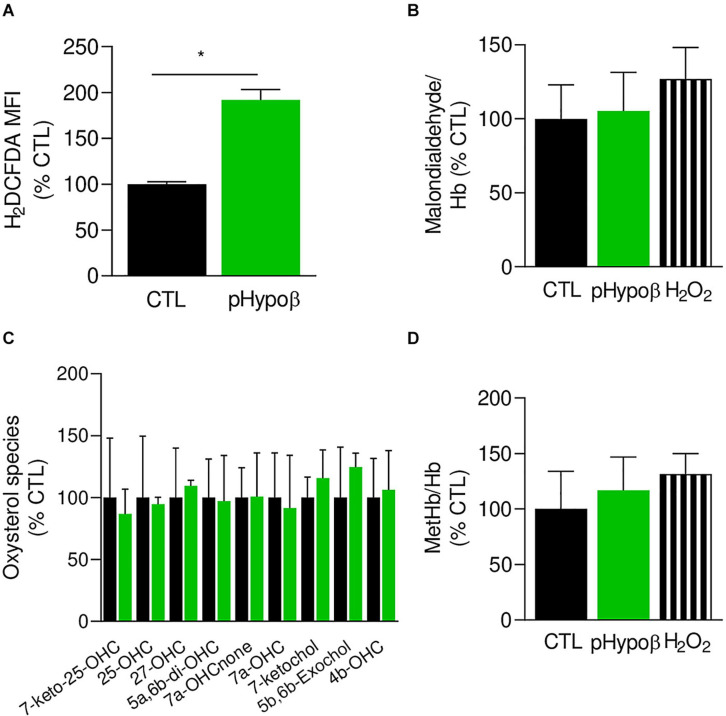
Accumulation of free reactive oxygen species in pHypoβ RBCs is not accompanied by lipid or hemoglobin oxidation. RBCs from healthy donors (black columns) or pHypoβ (green columns) were evaluated for free reactive oxygen species (ROS) accumulation **(A)**, lipid peroxidation **(B)**, oxysterol content **(C)**, and methemoglobin (MetHb) accumulation **(D)**. Hydrogen peroxide was used as positive control in **(B,D)** (hatched columns). All data are expressed as percentage of healthy untreated RBCs. **(A)** Intracellular ROS accumulation. RBCs were labeled with the non-fluorescent H_2_DCFDA which is transformed into fluorescent DCF inside the RBCs after de-esterification and interaction with ROS. Flow cytometry analysis allowed to determine the MFI of the global RBC population. Data are means ± SEM of 3–6 independent experiments. Mann–Whitney test; *p < 0.05. **(B)** Lipid peroxidation. Malondialdehyde (MDA), one final product of lipid peroxidation, was detected through interaction with thiobarbituric acid forming a fluorescent adduct. MDA levels were normalized to Hb content and data are means ± SD of triplicates from 2 independent experiments. **(C)** Membrane content in oxysterols. RBCs were washed, lysed, extracted for lipids and determined for 7-Keto-25-hydroxycholesterol (7-keto-25-OHC), 25-hydroxycholesterol (25-OHC), 27-hydroxycholesterol (27-OHC), 5α,6β-dihydroxycholesterol (5α,6β-diOHC), 7α-hydroxycholestenone (7α-OHCnone), 7α-hydroxycholesterol (7α-OHC), 7-ketocholesterol (7-ketochol), 5β,6β-epoxycholesterol (5β,6β-exochol) and 4β-hydroxycholesterol (4β-OHC). Results are expressed as percentage of control RBCs and are means ± SD of 2 independent experiments. **(D)** MetHb content. MetHb was determined using a sandwich Elisa and reported to the global Hb content. Data are means ± SD of triplicates from 1 experiment.

As the proportion of long chain PUFAs was decreased, we investigated whether pHypoβ RBCs could suffer from oxidative stress due to low circulating vitamin E levels (Supplementary Table 1), eventually leading to lipid and protein oxidation. We started by measuring ROS using H_2_DCFDA. A significant twofold increase of free ROS was observed in pHypoβ RBCs (Figure 5A) but not in a healthy donor without spleen (Supplementary Figure 1C), indicating that the ROS increase in pHypoβ was not due to splenectomy.

The extent of lipid peroxidation, determined through the level of MDA, was not modified in pHypoβ RBCs (Figure 5B). This might seem surprising because increased lipid peroxidation could be detected in HDL and platelets of patients with abetalipoproteinemia (Calzada et al., 2013). Nevertheless, only a slight increase was observed even for healthy RBCs treated with H_2_O_2_ (Figure 5B) and might be explained by the fact that MDA is not the only end product of lipid peroxidation. Nevertheless, oxysterol species were also maintained at levels similar to those found in healthy RBCs (Figure 5C).

Besides lipids, Hb is also a major target of oxidative stress in RBCs, generating metHb and eventually hemichromes (Mohanty et al., 2014). By ELISA we showed that pHypoβ RBCs exhibited a very slight metHb increase, comparable to the one obtained upon treatment of healthy RBCs with H_2_O_2_ (Figure 5D). All those data suggested that, despite major morphological changes and increase of free ROS, no obvious alterations in lipid and Hb oxidation appeared to occur in pHypoβ RBCs.

### pHypoβ RBCs Are Altered for Their Spectrin Network, Showing Either a Lower Density or a Patchy or Vesiculated Pattern

**FIGURE 6:**
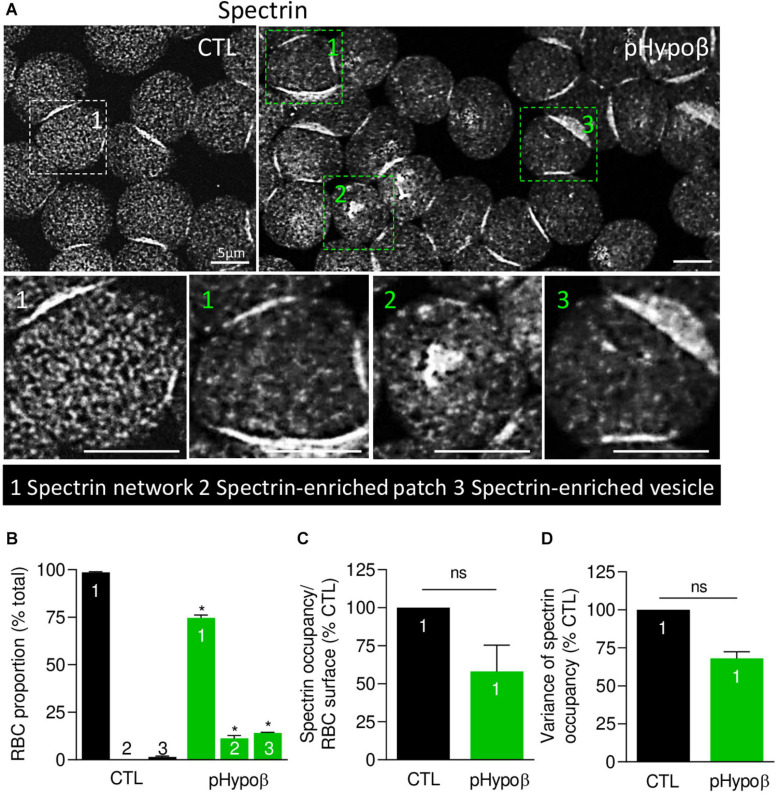
The spectrin network is altered in pHypoβ RBCs, showing either a lower density or a patchy or vesiculated pattern. RBCs from healthy donors (CTL, black) or pHypoβ (green) were spread onto PLL-coated coverslips, fixed/permeabilized, immunolabelled for spectrin and visualized by confocal fluorescence microscopy using the same settings for sample illumination. **(A)** Representative general views and zooms of RBCs with homogenous spectrin network (1), spectrin-enriched patches (2) and spectrin-enriched vesicles (3). **(B)** Quantification of the relative proportion of RBCs with homogenous spectrin network (1), spectrin-enriched patches (2) or spectrin-enriched vesicles (3). Data are means ± SEM of 3–6 independent experiments. Mann–Whitney tests to compare each RBC population in healthy vs. pHypoβ RBCs. *p < 0.05. **(C,D)** Quantification of spectrin occupancy normalized to the RBC surface **(C)** and variance of the spectrin labeling **(D)** in RBCs with homogenous spectrin network (population 1). Data are expressed as percentage of CTLs and are means ± SEM of 3 independent experiments. Mann–Whitney tests; ns, not significant.

We next evaluated by immunofluorescence the distribution of spectrin, another major target of oxidative stress (Voskou et al., 2015). Although the spectrin network was homogeneous in ∼99% of healthy RBCs, this proportion was significantly reduced to ∼75% in pHypoβ (population 1 at Figures 6A,B). Closer examination of this population indicated a tendency to decrease of the spectrin occupancy per RBC surface combined with a lower variance of spectrin occupancy (Figures 6C,D), suggesting that the spectrin network at the surface of pHypoβ RBCs was less dense than in healthy RBCs. Besides, the patient exhibited two additional populations with differential spectrin patterns, namely spectrin-enriched patches and vesicles (populations 2 and 3 at Figure 6A). The proportion of those two populations together increased by ∼25-fold as compared to healthy RBCs (populations 2 and 3 at Figure 6B). In conclusion, the spectrin cytoskeleton was altered in pHypoβ RBCs, ∼25% of them showing a patchy and vesiculated pattern and the remaining ∼75% exhibiting a less dense network.

### RBC Whole Stiffness and Curvature in Low and High Curvature Areas Are Increased in pHypoβ

**FIGURE 7:**
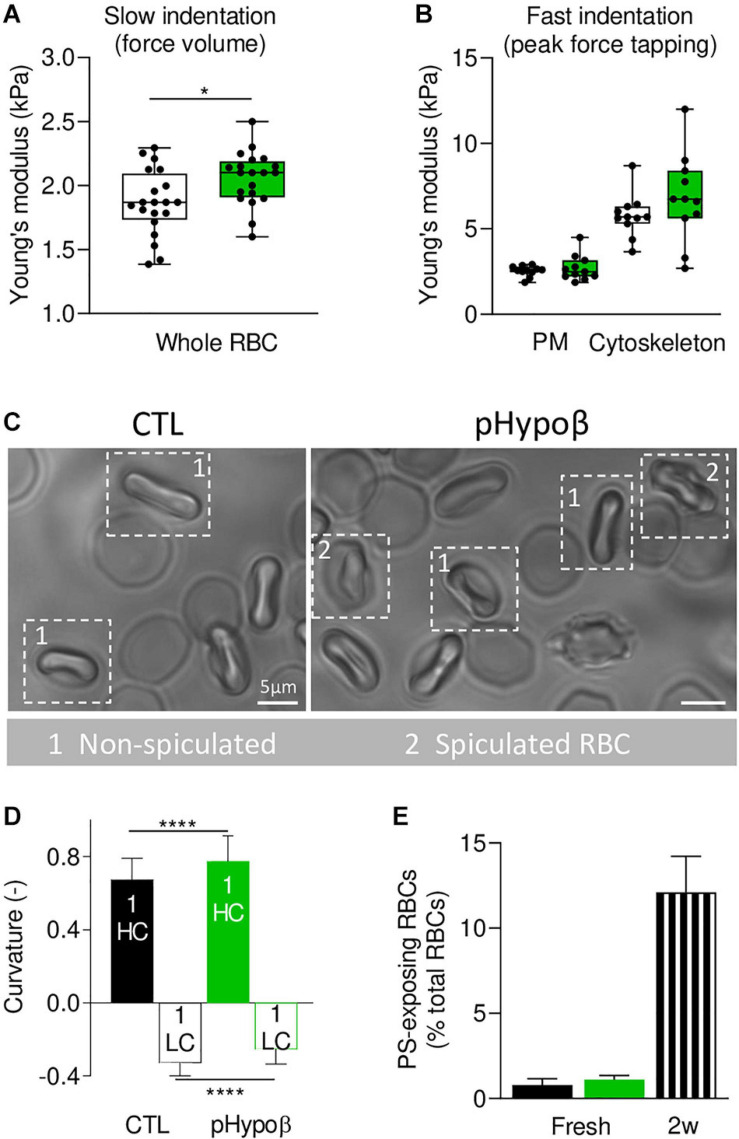
Membrane stiffness and curvature are increased in pHypoβ RBCs whereas membrane transversal asymmetry is not modified. RBCs from healthy donors (CTL, black) or pHypoβ (green) were evaluated for membrane stiffness **(A,B)**, curvature **(C,D)** and transversal asymmetry **(E)**. **(A,B)** Membrane stiffness. **(A)** Young’s modulus values extracted for CTL and pHypoβ RBCs in Slow indentation experiments, where the whole RBC mechanical behavior was analyzed. **(B)** Young’s modulus of CTL and pHypoβ RBCs obtained in Fast indentation experiments, where the elastic contribution of the plasma membrane (PM) and cytoskeleton were evaluated separately. Each data point represents the mean Young’s modulus value calculated for one RBC. Box plots depict 25th–75th percentiles, horizontal lines and cantered squares show mean values and error bars indicate SD. 20 RBCs were analyzed in **(A)** and 11 RBCs in **(B)**. Two-sample *t*-test. *p < 0.05. **(C,D)** Membrane curvature. RBCs were diluted, dropped off in IBIDI chambers and immediately observed by microscopy. Spiculated RBCs (indicated by 2) were distinguished from non-spiculated RBCs (indicated by 1) and the latter were then quantified for curvature in high curvature maxima (HC) and low curvature maxima (LC). 110 RBCs were analyzed for healthy donors and 50 RBCs for pHypoβ. Two-sample *t*-test. ****p < 0.0001. **(E)** Membrane transversal asymmetry. RBCs were labeled with fluorescent Annexin-V and then analyzed with FlowJo to determine the proportion of phosphatidylserine (PS)-exposing cells by positioning the cursor at the edge of the healthy fresh RBC population. RBCs from blood stored for 2 weeks at 4°C (2w) were used as positive control. Data are means ± SD of triplicates from 1 experiment.

To determine whether spectrin cytoskeleton modifications were accompanied by altered membrane biophysical properties, we analyzed RBC membrane stiffness and curvature. AFM at SLOW indentation revealed that pHypoβ RBCs were stiffer than healthy RBCs (Figure 7A). Since our previous experiments on RBCs treated with cytoskeleton-depolymerizing drugs using the same indentation approach revealed changes in the Young’s modulus, we inferred that the main contribution in the whole Young’s modulus originated from the cytoskeleton even if some contribution from the cytoplasmic viscosity cannot be discarded. To further test the contribution of the cytoskeleton in the whole RBC Young’s modulus vs. the plasma membrane stiffness, RBCs were analyzed at Fast indentation. Plasma membrane elasticity was similar in CTL and pHypoβ RBCs, while a stiffening of the cytoskeleton was observed for pHypoβ RBCs (Figure 7B). Hence, a higher variability of measurements was observed for the patient than healthy donors, which might reflect the presence of both acanthocytes and discocytes that were difficult to discriminate after RBC spreading. Interestingly, for both control and pHypoβ RBCs, the highest plasma membrane elastic modulus corresponded to the highest cytoskeleton stiffness (Supplementary Figure 2), suggesting that in diseased RBCs both the plasma membrane and the cytoskeleton were altered.

Because it was not possible to discriminate between spiculated and non-spiculated RBCs in AFM due to spreading we then used chambers compatible with RBC morphology preservation and quantified the membrane curvature of the non-spiculated RBCs (Figure 7C, population 1). Although the differential curvature between high (HC) and low curvature (LC) areas was preserved in the patient, curvature in both HC and LC areas was increased (Figures 7C,D). This latter increase was in good agreement with the lower spectrin occupancy per RBC surface in pHypoβ. Of note, no modification of membrane curvature was detected for RBCs from a healthy splenectomised donor (Supplementary Figure 1D). Altogether our data indicated that the cytoskeleton alteration in pHypoβ RBCs was accompanied by increased stiffness and curvature. To further analyze the potential alteration of the plasma membrane in the disease, we determined the membrane asymmetry both at the transversal and lateral levels.

### The Proportion of RBCs Exhibiting Phosphatidylserine Surface Exposure Is Preserved in pHypoβ

RBC PS exposure, a measure of membrane transversal asymmetry integrity loss, was not increased in pHypoβ RBCs as compared to healthy RBCs. As internal control, we used blood stored for 2 weeks in K^+^/EDTA tubes at 4°C, which we previously validated as a model of accelerated RBC aging *in vitro* based on morphological, biophysical and biochemical storage lesions, including PS surface exposure (Cloos et al., 2020). Thus, according to our previous work, a strong increase of PS surface exposure was observed in this condition (Figure 7E).

### Sphingomyelin-Enriched Domains at the pHypoβ RBC Surface Increase in Abundance and Do Not Respond to Intracellular Calcium Depletion

**FIGURE 8:**
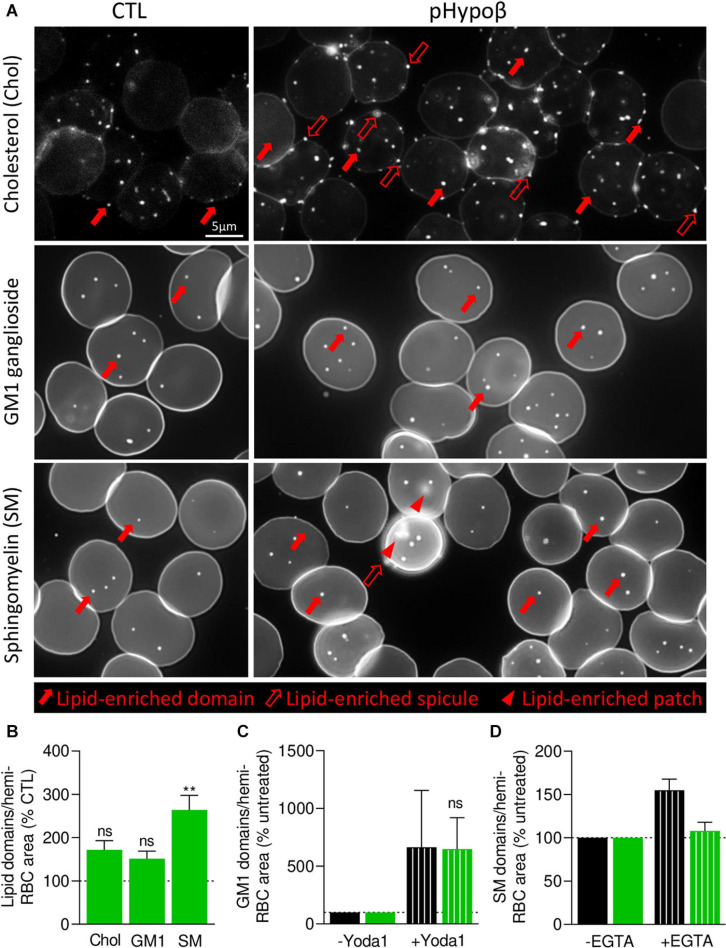
In contrast to cholesterol- and GM1-enriched domains, those enriched in sphingomyelin are modified in abundance and functionality at the pHypoβ RBC surface. RBCs from healthy donors (CTL, black columns) or pHypoβ (green columns) were either left untreated (not-hatched columns) or incubated with Yoda1 for 30 s (hatched columns in **C**) or EGTA for 10 min in a calcium-free medium (hatched columns in **D**). All RBCs were then either labeled with the fluorescent Theta toxin fragment specific to endogenous cholesterol and then immobilized on PLL-coated coverslips (Chol; **A,B**); or immobilized on PLL-coated coverslips and then labeled with fluorescent BODIPY analogs of GM1 ganglioside (GM1; **A–C**) or sphingomyelin (SM; **A,B,D**). All coverslips were then directly observed by vital fluorescence microscopy. **(A)** Representative images of Chol-, GM1-, and SM-enriched domains, spicules or patches in untreated RBCs. Large filled arrows, lipid-enriched domains; large open arrows, lipid-enriched spicules; arrowheads, lipid-enriched patches. **(B–D)** Quantification of lipid domain abundance in RBCs either untreated or treated with Yoda1 or EGTA to, respectively, activate PIEZO1 **(C)** or induce intracellular calcium depletion **(D)**. In **(D)** the calcium-free medium containing EGTA is maintained all along the experiment. Data are normalized to the hemi-RBC area and are means ± SEM of 4–5 independent experiments **(B)** or means ± SD/SEM of 2–3 independent experiments **(C,D)**. Kruskal–Wallis test followed by Dunn’s comparison test **(B)** and Wilcoxon matched-pairs signed rank tests **(C)**. ns, not significant; **p < 0.01.

We then took benefit from our expertise in submicrometric lipid domain organization at the RBC outer plasma membrane to evaluate the respective abundance of cholesterol-, GM1- and sphingomyelin-enriched domains, which contribute to the RBC deformation process (Leonard et al., 2017b; Conrard et al., 2018). Indeed, global quantitative analyses at the whole cell level may not reveal more localized alterations of acanthocyte membranes. Moreover, lipid domain abundance depends on membrane:cytoskeleton anchorage (Conrard et al., 2018). To visualize lipid domains, RBCs were labeled with a mCherry-toxin fragment specific to endogenous cholesterol or BODIPY fluorescent analogs of GM1 and sphingomyelin. Although the three types of lipid domains showed a tendency to increase in pHypoβ, only the abundance of sphingomyelin-enriched domains per hemi-RBC was significantly increased by 2.5-fold (Figures 8A,B). In contrast, no modifications of lipid domain abundance could be observed for RBCs from healthy splenectomised donors (Supplementary Figure 1E).

As GM1- and sphingomyelin-enriched domains were proposed to be implicated in calcium exchanges necessary for RBC deformation, we investigated their functionality in pHypoβ by stimulation of RBCs with Yoda1 and EGTA in a calcium-free medium to induce calcium entry and intracellular depletion, respectively. RBC incubation with Yoda1 led to an increased abundance of GM1-enriched domains in healthy RBCs, as previously shown (Conrard et al., 2018), and the same amplitude of response could be observed for pHypoβ RBCs (Figure 8C). In contrast, after intracellular calcium depletion, a heightened abundance of sphingomyelin-enriched domains was detected for healthy RBCs, as expected (Conrard et al., 2018), but not for pHypoβ RBCs (Figure 8D).

### pHypoβ RBCs Show Low Curvature-Associated Ceramide-Enriched Patches and High Curvature-Associated Cholesterol-Enriched Spicules

**FIGURE 9:**
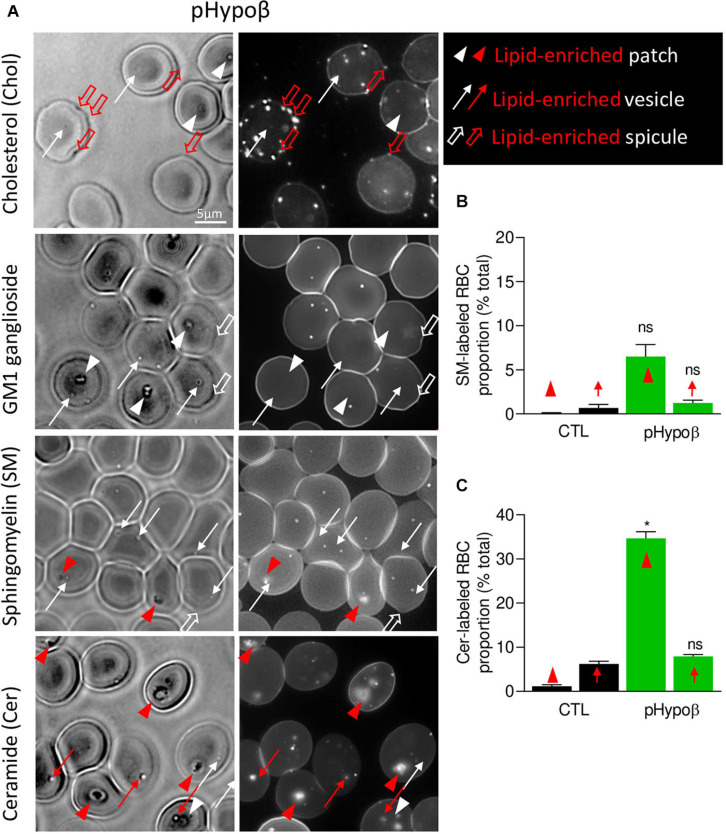
The spicules at the pHypoβ surface are mainly enriched in cholesterol whereas the patches and vesicles are mainly enriched in ceramide. RBCs from healthy donors (black columns) or pHypoβ (green columns) were either labeled with the fluorescent Theta toxin fragment specific to endogenous cholesterol and then immobilized on PLL-coated coverslips (Chol; **A**) or immobilized on PLL-coated coverslips and then labeled with fluorescent BODIPY analogs of GM1 ganglioside (GM1; **A**), sphingomyelin (SM; **A,B**); or ceramide (Cer; **A,C**). All coverslips were then directly observed by vital fluorescence microscopy. **(A)** Representative images of pHypoβ RBCs. Left, transmission microscopy image; right, fluorescence microscopy image. Lipid-enriched (red) or non-enriched (white) patches (arrowheads), vesicles (arrows), spicules (large open arrows) detected on the RBC surface on transmission images. **(B,C)** Quantification of the proportion of RBCs presenting SM- **(B)** or cer- **(C)** enriched patches or vesicles. Data are expressed as means ± SEM of 3–6 independent experiments. Mann–Whitney tests to compare enriched structures at the surface of healthy vs. pHypoβ RBCs; ns, not significant; *p < 0.05.

Besides well-defined submicrometric lipid domains, larger lipid-enriched patches (arrowheads at Figure 8A) and peripheral spicules (open arrows at Figure 8A) were seen in pHypoβ RBCs. We quantified the proportion of those lipid-enriched patches and spicules and their potential relationship with the patches and vesicles observed by contrast phase microscopy (see Figure 1D). Although none of the patches were enriched in cholesterol or GM1 ganglioside (white arrowheads at Figure 9A), some sphingomyelin-enriched patches were observed (red arrowheads at Figure 9A). However, their abundance was non-significantly different from the one in healthy RBCs (Figure 9B) and represented a low proportion of the patches evidenced at Figure 1D.

Intrigued by the increased content of ceramide and dihydroceramide species in pHypoβ (Figure 4B), we wondered if the patches could be enriched in ceramide. A ∼30-fold increase of the number of RBCs presenting ceramide-enriched patches was observed (red arrowheads at Figure 9A and quantification at Figure 9C). A heightened proportion of RBCs with ceramide-enriched patches was also observed for a healthy splenectomised donor but those RBCs accounted only for ∼10% of all RBCs compared to ∼35% for pHypoβ (Figure 9C and Supplementary Figure 1F). This could suggest that those patches are normally eliminated by the spleen.

Besides patches, ceramide-enriched vesicles can also be found but they did not significantly increase in abundance as compared to healthy RBCs (Figure 9C). The same was true for sphingomyelin-enriched vesicles (Figure 9B). In contrast, cholesterol-enriched spicules were observed at the edges of pHypoβ RBCs. As for patches, GM1 was enriched neither in vesicles nor in spicules (Figure 9A, white thin and thick arrows).

### pHypoβ RBCs Do Not Represent an Accelerated Model of RBC Aging

**FIGURE 10:**
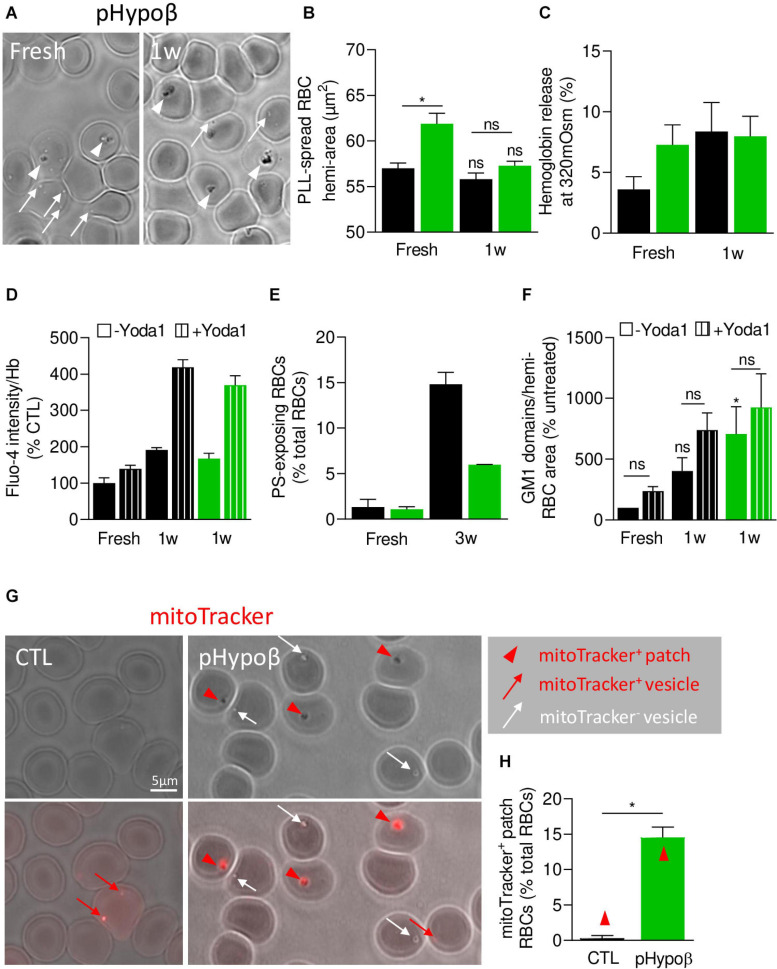
pHypoβ RBCs partly resist to RBC aging upon blood storage in K^+^/EDTA tubes at 4°C but exhibit a high abundance of residual mitochondrial fragments. RBCs from healthy donors (black columns) or pHypoβ (green columns) were either stored for 1–3 weeks at 4°C (1-3w) and assessed for RBC morphology and surface area **(A,B)**, osmotic fragility **(C)**, calcium content **(D)**, PS surface exposure **(E)** and GM1-enriched domain abundance **(F)**; or freshly analyzed for the presence of mitochondria remnants **(G,H)**. **(A–F)** RBC morphology and functionality upon storage at 4°C. **(A)** Morphology of fresh and 1 week-stored pHypoβ RBCs immobilized on PLL-coated coverslips as in Figure 1D. Images are representative of 3 experiments. **(B)** RBC surface determined as in Figure 1I. Data are means ± SEM of 3–7 independent experiments. Wilcoxon matched-pairs signed rank tests and Mann–Whitney test; ns, not significant; *p < 0.05. **(C)** RBC fragility determined as in Figure 2A. Data are means ± SD of triplicates from 1 experiment. **(D)** RBC calcium content. RBCs were labeled with Fluo4-AM as in Figure 2F, incubated with Yoda1 (hatched columns) and analyzed by fluorimetry. Data are normalized to Hb content and are means ± SD of triplicates from 1 experiment. **(E)** RBC PS exposure assessed as in Figure 7E. Data are means ± SD of triplicates from 1 experiment. **(F)** GM1-enriched domain abundance on RBCs at resting state and upon stimulation with Yoda1 (hatched columns), determined as in Figure 8C. Data are means ± SEM of 3 independent experiments. Kruskal–Wallis test followed by Dunn’s comparison test and Wilcoxon matched-pairs signed rank tests for the effect of Yoda1. ns, not significant; *p < 0.05. **(G,H)** RBC evaluation for the presence of mitochondrial remnants. RBCs were incubated for 30 min with a fluorescent mitoTracker, washed, dropped on PLL-coated coverslips and immediately observed by fluorescence microscopy. **(G)** Representative images. Upper panels, transmission; lower panels, transmission with fluorescence. Red arrowheads, mitoTracker-positive patches; red arrows, mitoTracker-positive vesicles; white arrows, mitotracker-negative vesicles. **(H)** Relative proportion of RBCs presenting mitoTracker-labeled patches as percentage of total RBCs. Data are means ± SEM of 3–6 independent experiments. Mann–Whitney test. *p < 0.05.

Altogether, our data indicated that pHypoβ RBCs were spiculated and showed enhanced ROS content, altered spectrin cytoskeleton integrity as well as increased membrane stiffness and curvature and abundance of sphingomyelin-enriched domains. These elements are consistent with the RBC morphological and biochemical storage lesions we previously revealed upon blood storage in K^+^/EDTA tubes for up to 4 weeks at 4°C as a model of accelerated RBC aging *in vitro* (Cloos et al., 2020). On the other hand, pHypoβ had increased membrane surface area and proportion of ceramide-enriched patches, rather consistent with reticulocyte properties (Ney, 2011). To therefore evaluate whether morphological, biochemical and biophysical alterations of pHypoβ RBCs could result from acceleration of RBC aging, RBCs from pHypoβ and healthy donors were stored for up to 3 weeks at 4°C and compared for morphology, fragility, functionality and biophysical properties. After 1 week of storage, the proportion of pHypoβ RBCs with patches did not increase any more (Figure 10A) and the differences in RBC membrane area and Hb release between pHypoβ and healthy donors seen in fresh RBCs were both abrogated (Figures 10B,C). The increase of intracellular calcium content measured after 1 week of storage in untreated and Yoda1-treated conditions were similar in pHypoβ and healthy donors (Figure 10D). Quite surprisingly, the extent of PS surface exposure measured after 3 weeks of storage increased three times less in pHypoβ than in healthy donors (Figure 10E). Regarding GM1-enriched domains, they increased in healthy RBCs after 1 week as expected from our previous data (Cloos et al., 2020), but this increase was even more pronounced and significant in pHypoβ RBCs. However, the increase of GM1-enriched domain abundance resulting from Yoda1 stimulation was lower in pHypoβ than healthy RBCs which might be due to the fact that GM1-enriched domain abundance was already higher at the surface of 1 week-stored pHypoβ RBCs than healthy RBCs stored for the same time (Figure 10F). We conclude that pHypoβ RBCs did not appear to represent an accelerated model of RBC aging.

### pHypoβ Shows a Very High Proportion of RBCs With Mitochondrial Fragments

We therefore evaluated the alternative possibility of a maturation defect of pHypoβ RBCs by labeling with a fluorescent mitoTracker. The proportion of pHypoβ RBCs presenting mitoTracker-positive patches at their surface increased by ∼40-fold as compared to healthy RBCs (Figures 10G,H). Importantly, the presence of those patches cannot be explained by the absence of spleen (Supplementary Figure 1G).

## Discussion

We here describe a case of familial hypobetalipoproteinemia, resulting from a heterozygosity for the pathogenic Gln845Argfs^∗^18 mutation in the *ApoB* gene. Similar to most patients with familial hypobetalipoproteinemia, pHypoβ presented reduced plasma cholesterol and ApoB levels, heightened plasmatic liver enzymes as well as a vitamin E level close to the lower reference limit (Clarke et al., 2006; Musialik et al., 2020). More surprisingly, a high proportion of acanthocytes was detected in this patient, most likely corresponding to the RBCs with surface patches and/or vesicles upon spreading on PLL-coated coverslips. Although those morphology changes were not accompanied by altered resistance to osmotic stress and cellular homeostasis, RBC deformability, oxidative stress, and membrane cytoskeletal and biophysical properties (curvature and stiffness) were impaired. Hence, a deeper imaging analysis revealed (i) heterogeneous spectrin cytoskeleton distribution in patches and vesicles; (ii) ceramide-enrichment and mitoTracker-partitioning in patches, suggesting the presence of mitochondria fragments resulting from RBC maturation defect; (iii) cholesterol-enrichment in spicules; and (iv) sphingomyelin-enriched domain increased abundance and decreased functionality.

### Extent of Acanthocytosis in Heterozygous Hypobetalipoproteinemia

Acanthocytes provide a typical clinical feature of abetalipoproteinemia, representing 50–90% of total RBCs (Biemer, 1980). In contrast, the proportion of acanthocytes and their existence are more debated for homozygous and heterozygous familial hypobetalipoproteinemia. In homozygous familial hypobetalipoproteinemia, while it has been stated that acanthocytes are as numerous as in abetalipoproteinemia Biemer (1980) and Tamura et al. (1988) reported two patients suffering from this disease with acanthocytes representing only 10–15% of all RBCs. For the heterozygous form of the disease, their presence is less frequently detected but not excluded (Biemer, 1980; Hardie, 1989; Clarke et al., 2006). Actually, splenectomy could be partly responsible for the unusual high proportion of spiculated RBCs detected for pHypoβ as (i) in some cases, acanthocytes can be observed post-splenectomy (Shah and Hamad, 2020); and (ii) the absence of the spleen in this patient could prevent hemolysis of acanthocytes normally provoked through splenic sequestration. Even if the post-splenectomy hypothesis could be supported by Howell–Jolly bodies observed on pHypoβ blood smears, it seems more likely that reduced RBC elimination as consequence of the absence of a spleen contributes to the abundance of acanthocytes in pHypoβ as no spiculated RBCs could be detected for a heathy splenectomised donor and acanthocytosis is part of the usual clinical picture of lipoprotein disorders and vitamin E deficiencies (Wong, 2004).

### Oxidative Stress, Cytoskeleton Defects and Membrane Stiffness Contribute to the RBC Phenotype in Heterozygous Hypobetalipoproteinemia

Vitamins E and A are important antioxidants and their supplementation is part of therapy for patients with homozygous familial hypobetalipoproteinemia and abetalipoproteinemia in order to prevent neurological and ophthalmological disorders (Granot and Kohen, 2004). This is not the case for heterozygous familial hypobetalipoproteinemia even if vitamin E levels below the normal range were detected (Burnett and Hooper, 2015). Regarding pHypoβ, although vitamin A was within the normal range, vitamin E was at the lower level. Clarke et al. actually detected reduced plasma vitamin E levels combined with reduced alpha-tocopherol content in RBCs of 9 patients suffering from heterozygous hypobetalipoproteinemia (Clarke et al., 2006). For patients suffering from abetalipoproteinemia and supplemented with liposoluble vitamins, observations differ. According to Granot and Kohen (2004), no signs for oxidative stress can be detected in the plasma of those patients while Calzada et al. (2013) reported decreased alpha-tocopherol levels in both platelets and HDLs of these patients combined with increased lipid peroxidation, consistent with the fact that vitamin E is a potent inhibitor of lipid peroxidation (Burnett and Hooper, 2015).

A high amount of free ROS was measured in pHypoβ RBCs but was not accompanied by increase in lipid peroxidation and metHb. However, it remains unclear where these ROS are coming from. Actually, vitamin K is not the typical antioxidant but it has nevertheless been shown to prevent oxidative stress in neurons (Li et al., 2003). Plasma levels of the latter were very low in pHypoβ. Taken together with low vitamin E levels, oxidative balance could be disturbed in pHypoβ RBCs because of reduced amount in circulating antioxidant vitamins eventually sufficient to avoid important lipid peroxidation but not to totally prevent RBC oxidative stress.

Besides membrane lipids and Hb, spectrin and several membrane:cytoskeleton anchorage proteins such as Band3 also represent targets of oxidative stress (Voskou et al., 2015). In pHypoß RBCs, we provided several lines of evidence supporting the role of the altered cytoskeleton in RBC morphology alteration. First, the abundance of RBCs with a homogenously dense spectrin network was decreased at the benefit of RBCs presenting spectrin-enriched patches and/or vesicles. Second, in the RBCs with an homogeneous spectrin network, the spectrin membrane occupancy was decreased, which could reflect a reduced membrane:cytoskeleton anchorage and lead to increased stiffness. Third, the latter increase in pHypoβ RBCs was confirmed by AFM.

The cytoskeleton alterations found in pHypoβ were in agreement with the literature. First, spectrin enrichment in the thorny projections of acanthocytes was already described by electron microscopy (Siegl et al., 2013). Second, spectrin modification through treatment with urea combined with lysophosphatidylcholine membrane insertion has been shown to induce the transformation of discocytes into acanthocytes (Khodadad et al., 1996). Third, cytoskeletal alterations and abnormalities of Band3 immunolabeling have been revealed in neuro-acanthocytosis (Wong, 2004; Adjobo-Hermans et al., 2015). Acanthocytes could be actually formed by alterations in Band3 conformation similar to echinocytes in which the ratio of outward and inward facing Band3 is responsible for the morphological alteration (Wong, 2004). Thus, spectrin but also Band3 could be the target of ROS in pHypoβ RBCs, leading to decreased membrane:cytoskeleton anchorage and to acanthocytes with spectrin-enriched projections. However, the role of Band3 in the disease remains to be explored.

### Alteration of Membrane Lipid Lateral Distribution Represents a New Contributor to the Acanthocytosis Seen in Heterozygous Hypobetalipoproteinemia

Analysis of the whole pHypoβ RBC population revealed no modification in the total cholesterol content in agreement with findings from other groups obtained on RBCs from patients with heterozygous hypobetalipoproteinemia (Biemer, 1980; Gheeraert et al., 1988). In abetalipoproteinemia and homozygous hypobetalipoproteinemia, observations are less consistent. Some groups reported increased cholesterol-to-phospholipid ratios (McBride and Jacob, 1970; Biemer, 1980; Barenholz et al., 1981) while others detected increased total cholesterol content without any increase in the cholesterol-to-phospholipid ratio (Iida et al., 1984) and still others described no alterations at all of the total cholesterol amount (Simon and Ways, 1964). Fatty acid composition was also largely maintained in pHypoβ RBCs, except for the slight increase of the linoleic and arachidonic acids, which could be related to specific dietary intake (Takkunen et al., 2013; Ford et al., 2016). To the best of our knowledge, the fatty acid content has not been studied in heterozygous hypobetalipoproteinemia. Abetalipoproteinemia and homozygous hypobetalipoproteinemia are instead generally associated with decreased linoleic acid content together with lower membrane fluidity. Besides, an increased sphingomyelin/lecithin or sphingomyelin/phosphatidylcholine ratio is also seen in RBCs of those patients (Ways et al., 1963; Simon and Ways, 1964; Cooper et al., 1977; Biemer, 1980; Barenholz et al., 1981; Iida et al., 1984). An increased sphingomyelin/phosphatidylcholine ratio resulting from a reduced abundance of phosphatidylcholine species was also evidenced in pHypoß. A higher (dihydro)ceramide content was also seen in pHypoβ RBCs in agreement with the increased ceramide-enriched patches. More surprisingly, a specific increase of lysophosphatidylethanolamine species was revealed while lysophosphatidylcholine is generally described to be associated with RBC morphological transformation into acanthocytes or echinocytes (Fujii and Tamura, 1984; Khodadad et al., 1996).

Lipid distribution at the RBC surface was analyzed in parallel to evaluate whether local changes in lipid domains could be revealed. We showed three types of membrane lipid distribution alterations. First, the abundance of sphingomyelin-enriched domains increased by 2.5-fold at the surface of pHypoβ RBCs. As membrane sphingomyelin content was not altered, the decreased membrane to cytoskeletal anchorage in pHypoβ RBCs could be responsible for this increase. This hypothesis is based on the observation by Conrard et al. that impairment of the membrane:cytoskeleton anchorage by PKC activation leads to an increase of sphingomyelin-enriched domains (Conrard et al., 2018). Second, ∼35 and ∼7% of pHypoβ RBCs presented ceramide- and sphingomyelin-enriched patches, respectively. Third, a high number of RBCs presented cholesterol-enriched spicules. The evident changes in membrane lipid organization stressed the importance of combining quantitative lipidomic analyses with qualitative lipid imaging to better understand diseases characterized by a variable- not systematically inventoried- amount of acanthocytes.

### RBC Functionality Is Poorly Affected in Heterozygous Hypobetalipoproteinemia

Quite surprisingly at first glance, only slight modifications of pHypoβ RBC functionality were observed. First, although no modification was seen in the poorly sensitive osmotic fragility test, osmotic gradient ektacytometry indicated a slight increase in surface area-to-volume ratio and therefore of osmotic fragility. Those observations were in accordance with the literature as for other models of acanthocytotic RBCs no modification in osmotic fragility is detected (Cooper, 1969; McBride and Jacob, 1970). Second, the intracellular calcium and ATP contents were normal. Third, the response of GM1-enriched domains to PIEZO1 activation through Yoda1 in fresh RBCs was similar in the patient and healthy donors.

Nevertheless, a deeper analysis indicated that the number of GM1-enriched domains was increased- although not significantly- in fresh RBCs and significantly in 1 week-old RBCs. Likewise, the abundance of sphingomyelin-enriched domains was enhanced and their response to intracellular calcium depletion through EGTA was abrogated. Thus, a fine analysis of the RBC surface both upon resting state and upon stimulation of calcium exchanges revealed functionality impairments in pHypoβ. Those alterations were not accompanied by altered calcium content or large increases in fragility probably because those measurements were made on the whole RBC population without any distinction between acanthocytes and discocytes. Interestingly, Ohsaka et al. reported on a patient with acute myelodysplasia with myelofibrosis who presented acanthocytes a heightened calcium uptake in RBCs combined with normal calcium levels (Ohsaka et al., 1989). In agreement with slight but detectable functionality impairment in pHypoβ, a loss of deformability after prolonged exposure to high shear stress was observed. Since this loss could only be partly explained by splenectomy, this observation revealed a slight reduction of membrane health of pHypoβ RBCs. This conclusion was further supported by alterations of RBC membrane biophysical properties.

### Defective RBC Maturation May Be a Novel Feature of Heterozygous Hypobetalipoproteinemia

In this study, we showed that the proportion of pHypoβ RBCs presenting mitoTracker-enriched patches, and thus mitochondrial fragments, increased by ∼40-fold as compared to healthy RBCs and represented ∼15% of total RBCs in pHypoβ. Moreover, ∼35% of pHypoβ RBCs presented ceramide-enriched patches. We propose that those ceramide-enriched patches corresponded to mitochondrial fragments, since (i) the increased abundance of those two types of patches in pHypoβ was similar; (ii) ceramides are known to be enriched in the outer mitochondrial membrane (Siskind, 2005); and (iii) mitoTracker and BODIPY-ceramide double-labeling in RBCs from patients with hereditary spherocytosis showed high colocalization (our unpublished data).

Besides the high number of mitochondria-positive RBCs, the RBC maturation defect in pHypoβ is further supported by (i) the increased membrane area measured after RBC spreading, normally reduced by vesiculation upon reticulocyte maturation (Trakarnsanga et al., 2017); (ii) the higher oxidative stress, as lipid peroxidation and activity of antioxidant enzymes are increased in reticulocytes as compared to mature RBCs (Sailaja et al., 2003); (iii) the increased abundance of GM1-enriched domains, as those domains are related to calcium influx in RBCs and calcium uptake is heightened in reticulocytosis (Ohsaka et al., 1989); and (iv) the threefold reduction in stored RBCs of surface exposure of PS, a lipid normally found in autophagic vesicles upon reticulocyte maturation (Trakarnsanga et al., 2017). Actually, the resistance to PS surface exposure has also been reported by Siegl et al. (2013) in chorea-acanthocytosis after RBC stimulation with lysophosphatidic acid. Thus, pHypoβ has a high proportion of acanthocytes which could result from a maturation defect during the R1 reticulocyte stage known to undergo significant rearrangements in reticulocyte membrane and intracellular components via several mechanisms including exosome release and mitophagy (Minetti et al., 2018, 2020).

### Experimental Strategy Strengths and Weaknesses

In this study, a wide range of research methods was used to evaluate morphological, biochemical and biophysical changes and their potential consequences for RBC functionality in hypobetalipoproteinemia. Moreover, thanks to a deep imaging analysis, we were able to reveal local changes in lipid composition and cytoskeleton distribution in acanthocytes. Nevertheless, our experimental strategy also presents some drawbacks that are inherent to the low prevalence of heterozygous hypobetalipoproteinemia with a high proportion of acanthocytes on one hand and to the study of membrane lipid lateral organization on the other hand. However, those limitations were minimized, based on the following evidences. First, although generalization of observations based on only one patient is difficult, our findings are in agreement with literature data on diseases associated with acanthocytosis. In fact, altered membrane composition and biophysical properties have also been demonstrated in a patient suffering from hereditary elliptocytosis and a series of patients suffering from hereditary spherocytosis, two diseases associated with increased membrane fragility due to cytoskeleton defects (Pollet et al., 2020; unpublished data), thereby supporting the importance of membrane:cytoskeleton interplay in RBC morphology and functionality. Second, although fluorescent lipid analogs were extensively used and validated in previous researches, their validity as *bona fide* surrogates of endogenous lipid counterparts is debated in view of the bulk fluorophore which can deeply modify biophysical properties. To minimize difficulties, we extensively validated all the lipid probes we used. For information regarding BODIPY-sphingomyelin and -GM1, we refer the reader to our previous papers (Tyteca et al., 2010; D’Auria et al., 2011, 2013; Carquin et al., 2016; Leonard et al., 2017b). For BODIPY-ceramide, the following lines of evidences supported a distribution as endogenous ceramides: (i) the fluorescent BODIPY-ceramide labeling correlated with the endogenous ceramide content measured by lipidomics both in pHypoβ and in RBCs upon blood storage at 4°C (Cloos et al., 2020); (ii) BODIPY-ceramide accumulates in the Golgi complex in nucleated cells (Tyteca et al., 2010) and in mitochondria remnants in RBCs from patients suffering from familial hypobetalipoproteinemia and from hereditary spherocytosis (data not shown), indicating that the lipid probe is able to join the intracellular compartments enriched in endogenous ceramide (Pagano et al., 1991); (iii) in the Golgi complex, BODIPY-ceramide can be efficiently converted into BODIPY-sphingomyelin, demonstrating the “one molecule at a time” conversion by the selectivity of the catalytic site of sphingomyelin synthase (Tyteca et al., 2010); (iv) BODIPY-ceramide accumulates in the inner plasma membrane leaflet, as revealed by resistance to back-exchange by BSA (Pollet et al., 2020) and demonstrating ability of the probe incorporated into the outer leaflet to flip to the inner one, as natural ceramide (Contreras et al., 2010); (v) BODIPY-ceramide-enriched domain abundance correlates with the ceramide content, as revealed by the decrease of ceramide-enriched domain abundance at the RBC membrane and the concomitant enrichment of ceramide species in RBC-derived extracellular vesicles at the end of the blood storage period in K^+^/EDTA tubes at 4°C (Cloos et al., 2020). Besides extensive validation of fluorescent lipid analogs, we developed the use of fluorescent Lysenin and Theta toxin fragments specific to endogenous sphingomyelin and cholesterol. However, it should be noted that, as fluorescent lipid analogs, toxin fragments also present drawbacks (Carquin et al., 2014, 2015, 2016) and it is therefore crucial to use complementary unrelated lipid probes to target one lipid, once possible. Third, regarding the imaging method used, although confocal microscopy offers several advantages such as vital imaging, multiple labeling and 3D-reconstruction, the size of observed lipid domains could have been overestimated.

### Conclusion and Overall Significance

In a case of familial hypobetalipoproteinemia, resulting from heterozygosity for the pathogenic Gln845Argfs^∗^18 mutation in the *ApoB* gene, we showed that the resulting acanthocytes exhibited impaired cytoskeleton and membrane biophysical properties without significant loss of RBC functionality as assessed by functional tests applied on the total RBC population, without any distinction between acanthocytes and discocytes (e.g., osmotic fragility test, intracellular calcium measurement). In contrast, more sensitive tests (e.g., ektacytometry) and assays aimed at investigating specific RBC populations (e.g., lipid domains, membrane curvature) did demonstrate functional impairments in pHypoβ.

Although our findings were generated from only one patient, the observed cytoskeleton and membrane alterations were consistent with literature data on diseases associated with acanthocytosis. Indeed, cytoskeletal alterations and abnormalities of Band3 immunolabeling have been shown in neuro-acanthocytosis (Wong, 2004; Adjobo-Hermans et al., 2015). The resistance to PS surface exposure has also been reported in chorea-acanthocytosis after RBC stimulation with lysophosphatidic acid (Siegl et al., 2013). The modest alterations of pHypoβ RBC functionality agreed with the absence of modification in osmotic fragility in other models of acanthocytotic RBCs (Cooper, 1969; McBride and Jacob, 1970).

From a more global point-of-view, altered membrane composition and biophysical properties have also been demonstrated in two RBC disorders associated with altered cytoskeleton function and a large diversity of RBC morphological changes and deformability alterations (Pollet et al., 2020; unpublished data), indicating the close membrane:cytoskeleton interplay and its role in RBC morphology and functionality. More specifically, a parallelism could be established with the patients with hereditary spherocytosis exhibiting a high proportion of spiculated RBCs (our unpublished data).

Our study demonstrates that evaluation of membrane biophysical properties and membrane lipid distribution could be of benefit in the diagnosis and better understanding of RBC disorders.

## Data Availability Statement

The original contributions generated for this study are included in the article/Supplementary Material, further inquiries can be directed to the corresponding author.

## Author Contributions

A-SC and DT designed the experiments, analyzed and interpreted the data, and wrote the manuscript. LGMD and JW identified the patient and established the diagnosis. MM, AS, and JV collected, analyzed, and quantified lipid imaging data. PVDS did the electron microscopy experiments. MR and RW performed ektacytometry measurements. EM and YL were responsible for fatty acid analysis while RT and GM performed lipidomics. ACD and DA generated and analyzed AFM data. All authors reviewed the final version of the manuscript.

## Conflict of Interest

The authors declare that the research was conducted in the absence of any commercial or financial relationships that could be construed as a potential conflict of interest.

## Supplementary Material

Supplementary Figure 1 Except for a slight increase in ceramide-enriched patches, RBCs from a healthy splenectomised donor exhibit similar morphology, biophysical properties, functionality and maturation than healthy non-splenectomised donors. RBCs from healthy non-splenectomised (+spleen; black columns) donors and a healthy splenectomised (−spleen; gray columns) donor were compared for RBC membrane area **(A)**, osmotic fragility **(B)**, ROS intracellular content **(C)**, curvature **(D)**, lipid domain abundance **(E)**, and proportion of RBCs with ceramide (cer)- or mitoTracker-enriched patches and vesicles **(F,G)**. **(A)** RBC membrane area assessed as in Figure 1I. Data are means ± SEM of 5 independent experiments. Mann–Whitney test. Ns, not significant. **(B)** RBC osmotic fragility determined as in Figure 2B. Data from 1 experiment. **(C)** ROS content evaluated as in Figure 5A. Data are means ± SD of triplicates from 1 experiment. **(D)** RBC curvature determined on RBCs in suspension as in Figure 2B. Representative images of 2 independent experiments. **(E)** Lipid domain abundance determined as in Figure 8B. Data are means ± SEM of 4–5 independent experiments. Kruskal–Wallis test followed by Dunn’s comparison test. Ns, not significant. **(F)** Proportion of RBCs with cer-enriched patches and vesicles determined as in Figure 9C. Upper panel, representative image of RBCs from the splenectomised donor; lower panel, quantification of cer-enriched patches (red arrowheads) and vesicles (red arrows) in RBCs from non-splenectomised (black columns) and splenectomised (gray columns) donors. Data are means ± SEM of 3–6 independent experiments. Mann–Whitney tests to compare non-splenectomised vs. splenectomised RBCs; ns, not significant; ^∗^*p* < 0.05. **(G)** MitoTracker labeling as in Figure 10G. Upper panels, transmission; lower panels, transmission combined with fluorescence. MitoTracker-positive vesicles are indicated with red arrows. Images are representative of 3 experiments.

Supplementary Figure 2 Relation between RBC plasma membrane and cytoskeleton elastic modulus in the fast indentation experiments. RBCs from healthy donors or pHypoβ were evaluated for both plasma membrane and cytoskeleton Young’s modulus. Each dot depicts one RBC measured for plasma membrane and cytoskeleton Young’s modulus. Experimental data are from Figure 7B.

Supplementary Table 1 pHypoβ clinical data. Clinical parameters were assessed for pHypoβ after blood collection. MCV, mean corpuscular volume; MCH, mean corpuscular hemoglobin; Gamma-GT, Gamma-glutamyltransferase; ASAT, aspartate transaminase; ALAT, alanine transaminase; LD, lactate dehydrogenase. Values upon or under the reference values are indicated in red or blue, respectively.
