# Erratum: Impaired Cytoskeletal and Membrane Biophysical Properties of Acanthocytes in Hypobetalipoproteinemia – A Case Study

**DOI:** 10.3389/fphys.2021.695193

**Published:** 2021-05-07

**Authors:** Frontiers Production Office

**Affiliations:** Frontiers Media SA, Lausanne, Switzerland

**Keywords:** acanthocytosis, lipidomics, lipid domains, membrane biophysical properties, reactive oxygen species, ceramide, mitochondria, erythropoiesis

Due to a production error, there was an error regarding the affiliation for “Andra C. Dumitru.” The correct affiliation should be “^5^ Louvain Institute of Biomolecular Science and Technology, UCLouvain, Ottignies-Louvain-la-Neuve, Belgium.” Also affiliation 5 was erroneously duplicated as affiliation 7. Consequently, affiliation for “David Alsteens” has been renumbered. The publisher apologizes for this mistake.

The original article has been updated.

