# Supplementary figures and images for: Impaired Cytoskeletal and Membrane Biophysical Properties of Acanthocytes in Hypobetalipoproteinemia – A Case Study
Source: Front Physiol. 2021 Feb 23;12:638027. doi: 10.3389/fphys.2021.638027 (PMC7940373; doi:10.3389/fphys.2021.638027)

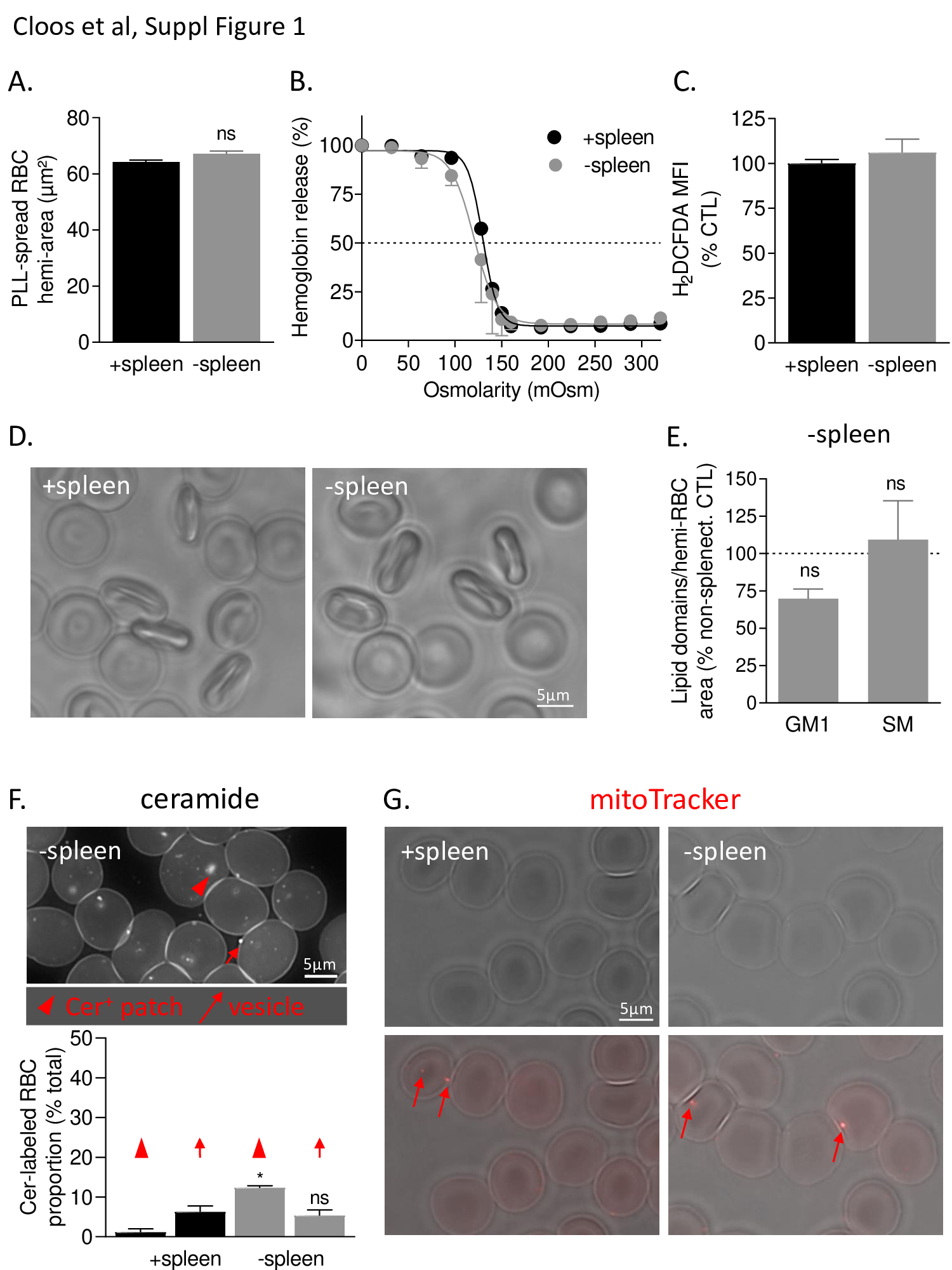

Supplement: Supplementary Figure 1 — Except for a slight increase in ceramide-enriched patches, RBCs from a healthy splenectomised donor exhibit similar morphology, biophysical properties, functionality and maturation than healthy non-splenectomised donors. RBCs from healthy non-splenectomised (+spleen; black columns) donors and a healthy splenectomised (−spleen; gray columns) donor were compared for RBC membrane area (A), osmotic fragility (B), ROS intracellular content (C), curvature (D), lipid domain abundance (E), and proportion of RBCs with ceramide (cer)- or mitoTracker-enriched patches and vesicles (F,G). (A) RBC membrane area assessed as in Figure 1I. Data are means ± SEM of 5 independent experiments. Mann–Whitney test. Ns, not significant. (B) RBC osmotic fragility determined as in Figure 2B. Data from 1 experiment. (C) ROS content evaluated as in Figure 5A. Data are means ± SD of triplicates from 1 experiment. (D) RBC curvature determined on RBCs in suspension as in Figure 2B. Representative images of 2 independent experiments. (E) Lipid domain abundance determined as in Figure 8B. Data are means ± SEM of 4–5 independent experiments. Kruskal–Wallis test followed by Dunn’s comparison test. Ns, not significant. (F) Proportion of RBCs with cer-enriched patches and vesicles determined as in Figure 9C. Upper panel, representative image of RBCs from the splenectomised donor; lower panel, quantification of cer-enriched patches (red arrowheads) and vesicles (red arrows) in RBCs from non-splenectomised (black columns) and splenectomised (gray columns) donors. Data are means ± SEM of 3–6 independent experiments. Mann–Whitney tests to compare non-splenectomised vs. splenectomised RBCs; ns, not significant; ∗p < 0.05. (G) MitoTracker labeling as in Figure 10G. Upper panels, transmission; lower panels, transmission combined with fluorescence. MitoTracker-positive vesicles are indicated with red arrows. Images are representative of 3 experiments. [file Image_1.JPEG]

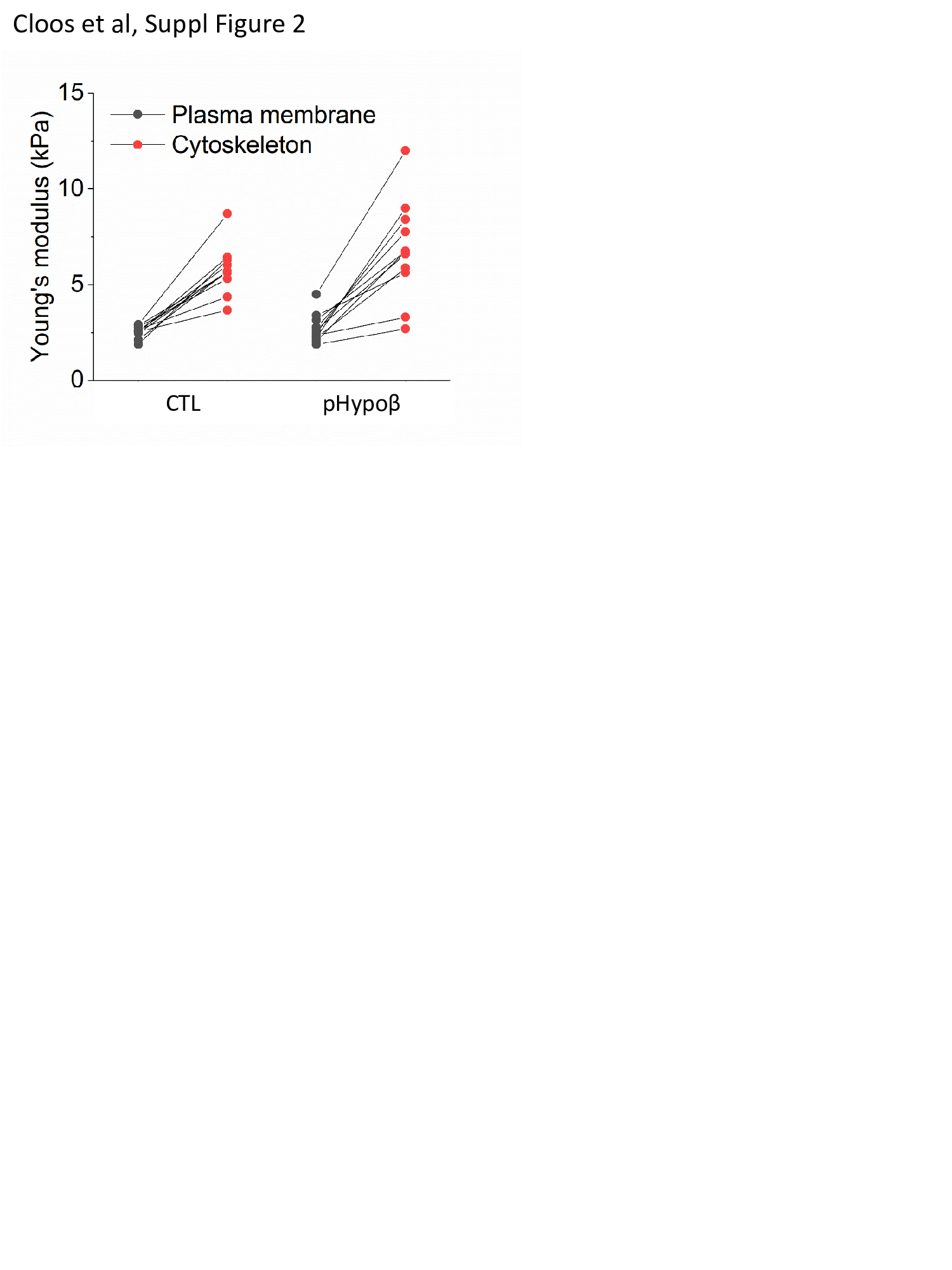

Supplement: Supplementary Figure 2 — Relation between RBC plasma membrane and cytoskeleton elastic modulus in the fast indentation experiments. RBCs from healthy donors or pHypoβ were evaluated for both plasma membrane and cytoskeleton Young’s modulus. Each dot depicts one RBC measured for plasma membrane and cytoskeleton Young’s modulus. Experimental data are from Figure 7B. [file Image_2.JPEG]
