# Supplementary material for: Impaired Cytoskeletal and Membrane Biophysical Properties of Acanthocytes in Hypobetalipoproteinemia – A Case Study
Source: Front Physiol. 2021 Feb 23;12:638027. doi: 10.3389/fphys.2021.638027 (PMC7940373; doi:10.3389/fphys.2021.638027)
Supplement: Supplementary Table 1 — pHypoβ clinical data. Clinical parameters were assessed for pHypoβ after blood collection. MCV, mean corpuscular volume; MCH, mean corpuscular hemoglobin; Gamma-GT, Gamma-glutamyltransferase; ASAT, aspartate transaminase; ALAT, alanine transaminase; LD, lactate dehydrogenase. Values upon or under the reference values are indicated in red or blue, respectively. [file Table_1.pdf]

Cloos et al, Suppl Table 1

	pHypoβ	Normal range	Units
Reticulocytes	59	25 - 120	* 10^9/L
MCV	92	80 - 97	fL
MCH	1.79	1.75 - 2.25	fmol
Gamma-GT	84	0 - 55	U/L
ASAT	54	0 - 35	U/L
ALAT	84	0 - 45	U/L
LD	287	0 - 250	U/L
Cholesterol	2.1	3.5 - 6.5	mmol/L
Triglycerides	0.3	0.0 - 2.0	mmol/L
Apo A1	1.26	1.1 - 2.05	g/L
Apo B	<0.3	0.55 - 1.40	g/L
Vitamin A	2.4	1 - 2.4	µmol/L
Vitamin E	14	12 - 46	µmol/L
Vitamin K	0.6	0.8 - 5.3	nmol/L
